# iCopyDAV: Integrated platform for copy number variations—Detection, annotation and visualization

**DOI:** 10.1371/journal.pone.0195334

**Published:** 2018-04-05

**Authors:** Prashanthi Dharanipragada, Sriharsha Vogeti, Nita Parekh

**Affiliations:** Center for Computational Natural Sciences and Bioinformatics, International Institute of Information Technology, Hyderabad, India; Oklahoma State University, UNITED STATES

## Abstract

Discovery of copy number variations (CNVs), a major category of structural variations, have dramatically changed our understanding of differences between individuals and provide an alternate paradigm for the genetic basis of human diseases. CNVs include both copy gain and copy loss events and their detection genome-wide is now possible using high-throughput, low-cost next generation sequencing (NGS) methods. However, accurate detection of CNVs from NGS data is not straightforward due to non-uniform coverage of reads resulting from various systemic biases. We have developed an integrated platform, *i*CopyDAV, to handle some of these issues in CNV detection in whole genome NGS data. It has a modular framework comprising five major modules: data pre-treatment, segmentation, variant calling, annotation and visualization. An important feature of *i*CopyDAV is the functional annotation module that enables the user to identify and prioritize CNVs encompassing various functional elements, genomic features and disease-associations. Parallelization of the segmentation algorithms makes the *i*CopyDAV platform even accessible on a desktop. Here we show the effect of sequencing coverage, read length, bin size, data pre-treatment and segmentation approaches on accurate detection of the complete spectrum of CNVs. Performance of *i*CopyDAV is evaluated on both simulated data and real data for different sequencing depths. It is an open-source integrated pipeline available at https://github.com/vogetihrsh/icopydav and as Docker’s image at http://bioinf.iiit.ac.in/icopydav/.

## Introduction

With the advent of high throughput sequencing techniques, there has been considerable interest in identifying population-specific structural variants (SVs), and their possible role in disease. Among various structural variations, copy number variations (CNVs) are shown to contribute significantly to human genome diversity and predisposition to disease. CNVs comprise deletions and duplications of DNA segments of size ≥ 50bp and maybe as large as 1Mbp, spanning about ~9.5% of human genome [1]. This may lead to alterations in protein-coding regions through gene dosage imbalance or gene disruption, or through changes in the expression of non-coding genes and regulatory elements.

Formation of CNVs is mainly driven by inaccurate repair and mistakes in replication. One major repairing mechanism of DNA double strand breaks (DSB) is homologous recombination. The repair mechanism may misalign the DSBs to another homologous region, resulting in non-allelic homologous recombination (NAHR) (aided by repeats and segmental duplications). This mechanism is usually observed in the formation of recurrent CNVs. Another repair mechanism at DSB sites involves non-homologous end joining (NHEJ) and microhomology-mediated end joining (MMEJ) and may lead to deletion/insertions (aided by repeats). Unlike NAHR, these mechanisms do not require any template for joining the DSBs. CNVs can also be generated due to mistakes in the replication process, *viz*., polymerase slippage and template switching. In template switching, DNA replication fork stalls, lagging strand disengage from the DNA and shifts to another replicating DNA fragment and restarts the process, termed as fork stalling and template switching (FoSTeS) (aided by repeats). This mechanism results in the generation of non-recurrent CNVs. Mobile element insertion (MEI) is a mechanism that allows transposable elements to make their copies and insert in new locations that are usually flanked with inverted repeats (mediated by retrotransposons, DNA transposons and endogeneous retroviruses) [2,3].

Traditional approaches such as karyotyping, fluorescence in situ hybridization (FISH) and array-based methods such as comparative genomic hybridization (arrayCGH) and single-nucleotide polymorphism (SNP) arrays, are now being replaced by high-throughput, next generation sequencing (NGS) based approaches, due to the reducing cost and higher resolution of these techniques. Various strategies used in the detection of CNVs in NGS data are: paired-end mapping (PEM), split-read (SR), depth-of-coverage (DoC), *de novo* assembly (AS) and their combinatorial methods. Each of these approaches has their own strengths and shortcomings, limiting the detection of the complete spectrum of CNVs by any particular approach. For instance, PEM and SR based approaches accurately detect small CNVs, however, these are not suitable for predicting insertions larger than the insert size and fail to identify CNVs in low complexity regions and segmental duplications. *De novo* assembly-based methods provide an unbiased approach to predict novel genetic variants as these methods are independent of reference based read alignment. However, these methods require read coverage ≥ 30× and are highly computationally demanding. The Depth-of-Coverage (DoC) based approaches are most popular due to their ability to determine the absolute copy number of the variant region in single-read data, but, suffer from precise breakpoint predictions of the CNV calls.

In this study we highlight various issues in DoC based approaches. These approaches are based on the assumption that read depth is approximately proportional to the copy number of the DNA region. However, due to various systemic biases, detection of CNVs is so not trivial. Two major biases affecting the read depth are GC bias and mappability bias [3]. Correcting for these two biases, called data pre-treatment, is an important step and various approaches have been proposed (summarized in Table 1). The GC content is not uniform along the human genome and PCR amplification step is biased resulting in fewer reads from low GC or high GC regions. This introduces a bias in correct estimation of read-depth (RD) signals in low or high GC regions, called GC bias. Filtering out low and high GC regions, normalizing the read depth in a bin based on its GC content (in absence of control sample), or taking the ratio of copy number in corresponding bins in case-control samples may aid in addressing this bias. Another major bias that affects CNV calling in DoC-based approaches is handling of ambiguous reads, called mappability bias. In the read alignment step, ambiguous reads may either be aligned to the best scoring position, *k*-best scoring positions, to all possible positions [4], or randomly assigned to any one of the possible positions [5,6]. This results in non-uniform read depth. The problem is more prominent in the case of short reads (36–150 bp). Choosing an appropriate threshold for filtering mappability regions, and/or normalizing reads in a bin with respect to the mappability score of the bin can help in addressing this bias. In this study, using *i*CopyDAV, we show that appropriate handling of GC bias and mappability bias methods is dependent on the bin size, type of CNVs to be detected and the sequencing coverage.

**Table 1:** Some of the popular data pre-treatment and segmentation methods in DoC-based approaches are listed.

Step	Method	Tool
**Data pre-treatment:** **GC bias correction**	Filter reads based on GC content score	Control-FREEC [7], OncoSNP-seq [8]
	Matched-control based ratio	CNV-seq [9], CNAseg [10], JointSLM [11], CNVrd2 [12]
	Loess regression	ReadDepth [13], cnvHiTSeq [14], CNAnorm [15], SMASH [16]
	Median approach	RDXplorer [17], CNVnator [18]
	Mean fragment count-based correction	GCcorrect [19], DeepTools [19]
	Quantile normalization	GROM-RD [20]
	Rolling median approach	CNVkit [21]
**Data pre-treatment: Mappability**	Ignore all multi-reads	BIC-seq2 [22], Segseq [23]
	Randomly assign reads	CNVnator [18]
	Filter reads based on mappability cut-off threshold	Control-FREEC [7], CONIFER [24]
	Normalize using mappability score	CNAseg [10], ReadDepth [13], HMMcopy [25]
**Segmentation**	Circular binary segmentation (Top-down)	ReadDepth [13], CNAnorm [15], SMASH [16]
	Mean-shift (Top-down)	CNVnator [18]
	Event-wise testing (Bottom-up)	RDXPlorer [17]
	Hidden Markov model (Bottom-up)	HMMcopy [25], MGP-HMM [26]
	Total variation minimization(Bottom-up)	CNV-TV [27]
	LASSO-regression (Top-down)	Control-FREEC [7]
	Shifting-level model (Bottom-up)	JointSLM [11]
	Convex hull-based model (Bottom-up)	CNV-CH [28]

Another major step in DoC based approach is segmentation. In this step, the objective is to cluster read depth signals of similar intensities, and those deviating from the mean read depth signal are identified as variant regions. Read depth signals are very noisy because of several factors: sequencing error, mapping error (multiple mapping, mismatching), presence of SNPs and indels. Major challenge in segmentation is to distinguish spurious variant regions from true copy variant regions. Various segmentation approaches have been proposed (summarized in Table 1) which can be majorly categorized as change-point detection algorithms followed by a merge procedure [13], or statistical hypothesis testing that assumes read-depth signal obeys a certain probability distribution (e.g., Poisson, negative-binomial) [9,11]. Using *i*CopyDAV, we show that these methods suffer from under-cut or over-cut problem and need to be judiciously chosen depending on the type and size of CNVs to be detected and the sequencing coverage of the data.

To analyze the functional impact of CNVs, information regarding their overlap with various functional elements, *viz*., protein-coding genes, non-coding RNAs, genomic regulatory elements (promoters and enhancers), etc., known CNVs and disease association is desirable. Though numerous independent annotation tools are available for functional annotation of CNVs (summarized in Table 2), in our knowledge no CNV detection tool has the annotation component. Visualization of CNVs is also an important feature, and aid in analyzing distribution of CNVs across the chromosome/genome and identify CNV clusters.

**Table 2:** Some of the popular annotation and visualization methods for CNVs are listed.

Step	Tool	Features
**Annotation**	CNVannotator [29]	Functional elements, Structural elements (SD), Known CNVs (DGV, dbVar), Clinical features (CNVD)
	DeAnnCNV [30]	Functional elements, Known CNVs (dbVar), Clinical features (ClinVar)
	SG-ADVISOR [31]	Functional elements, Known CNVs (DGV, Scrips-Wellderly Genome, 1000 Genome project), Clinical features (OMIM, ClinVar, HGMD)
	cnvScan [32]	Functional elements, Known CNVs (DGV, 1000 Genome project), Clinical features (OMIM, ClinVar, DECIPHER, ExAC)
	ANNOVAR [33]	Functional elements, Structural elements (SD)
**Visualization** (Integrated with tool)	Control-FREEC [7]	Complete chromosome view	
	CNAnorm [15]	Complete chromosome view	
	ReadDepth [13]	Complete chromosome view, GC bias correction	
**Visualization** (Standalone)	CNView (GenVisR) [34]	Complete chromosome view, specific coordinates along with the gene annotations from UCSC genome browser	
	cnvCurator [35]	Interactive visualizer, Editing the CNV breakpoints	
	cnvKit [21]	Complete chromosome view, User defined coordinates, across the samples	

SD: Segmental duplications, DGV: Database of Genomic Variants, CNVD: Copy number variation in Disease, dbVar: Database of Genomic Structural Variants, HGMD: Human Gene Mutation Database, OMIM: Online Mendelian Inheritance in Man, DECIPHER: Database of Genomic Variation and Phenotype In Humans using Ensembl Resources, ExAC: Exome Aggregation Consortium

Previous studies [36,37] have shown that using multiple methods may improve the reliability of CNV prediction compared to any single approach. Integrated platforms have been developed that incorporate either different strategies *viz*., PEM, SR, DoC methods (e.g., CNVer [38], ERDS [39] and Clever [40]) or merge output from various tools that are based on different strategies (e.g., HugeSeq [41], SVMerge [42] and MetaSV [43]). Though these platform perform better than single approaches, these do not provide user the flexibility to choose the type of approach (es). Also, most of these platforms do not provide annotation and visualization features. Thus, there clearly exists a need for an end-to-end pipeline providing both variant calling and annotation to the predicted variants on a single platform. With this objective we developed an integrated platform, *i*CopyDAV, for CNV detection in whole genome NGS data based on DoC approaches. It has a modular framework and comprises five major steps: Data pre-treatment (handling GC bias and mappability bias), Segmentation (divisive and agglomerative approaches), CNV calling (type and copy number), Annotation (functional elements, known CNVs, clinical associations and structural features) and Visualization (GC profile and CNV distribution plots). It is different from various integrated platforms in that it provides the user with various options at every step of the pipeline: starting from choosing appropriate bin size to methods for removing GC and mappability biases, and segmentation, and provides an end-to-end solution with its annotation and visualization features. Thus, in *i*CopyDAV, the users can customize their workflow depending on the type and size of the CNVs to be detected and the sequencing coverage of the data. If desired, the user can also merge CNV calls from various workflows (union or intersection) in *i*CopyDAV. An important feature of *i*CopyDAV is its computational efficiency due to parallelization of the segmentation algorithms incorporated enabling faster detection of CNVs even in high sequence coverage whole genome data.

## Materials and methods

**Fig 1:**
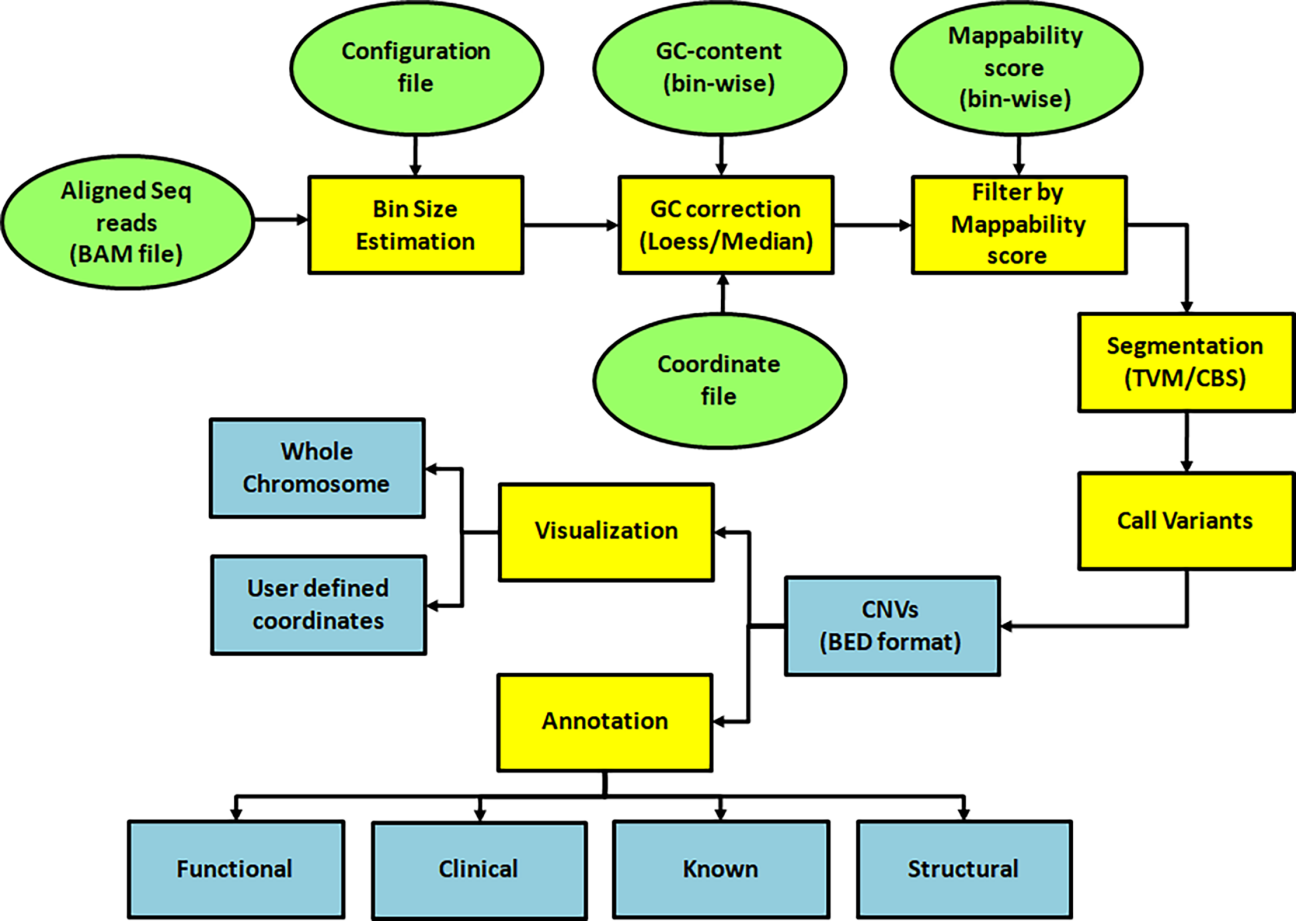
Flowchart of the *i*CopyDAV pipeline (input from user: ‘green’, computational steps: ‘Yellow’, Output: ‘Blue’).

*i*CopyDAV is an open-source pipeline implemented in a combination of programming languages (C++, R and bash) which are easy to understand and modify. The flowchart of the proposed CNV detection pipeline is given in Fig 1. Aligned sequence reads in BAM format is the primary input to the program. Additionally, files containing bin-wise GC content and mappability scores for all chromosomes need to be provided for data pre-treatment. For human reference genome assemblies (hg18/hg19), read length = 100 bp and default bin-size (100 bp), these files are made available at http://bioinf.iiit.ac.in/icopydav/. Step-by-step instruction for generating these files for any bin-size is given in the tutorial along with a sample configuration file. The pipeline doesn’t require matched-control sample or paired-end reads and can be used for CNV detection in samples from species other than human (however, functional annotation and visualization is available only for human genome assembly hg18/hg19). The pipeline has a modular framework and is command-line based, offering the user flexibility in configuring it as per their requirement. *i*CopyDAV is freely available at GitHub (https://github.com/vogetihrsh/icopydav) and can also be accessed via Docker’s container image at http://bioinf.iiit.ac.in/icopydav/. In this case the user need to first install Docker on the local machine and then run the pipeline; all the dependencies will get installed automatically (instructions provided in the tutorial).

### 1. Data preparation

The first step in any depth of coverage based approach is to compute the read depth profile along the length of the chromosome. This is typically computed over an appropriate window or bin size, instead of at every base, to reduce random fluctuations and obtain a smoothed read depth profile. For this the reference genome is binned into non-overlapping windows and the read count in each bin, called its read depth (RD) value, is computed. Bin size is a very crucial parameter in accurate detection of CNVs and is dependent on the sequencing coverage; low sequence coverage data requiring larger bin size for reliable CNV prediction. Since smaller bin size would result in smaller breakpoint error, high sequence coverage data is required for accurate detection of small CNVs (≤ 1Kbp) [17]. In *i*CopyDAV, the user may set the bin size judiciously depending on the sequence coverage and the size of the CNVs to be detected (default 100 bp) (~ 500 bp for low (4–6×), ~100 bp for medium (20–30×) and ~ 30 bp bin size for very high coverage data (~ 100×) [18]). Alternately, one may use the function ***calOptBinSize*** to obtain an optimal bin size for a given sequencing coverage using the expression λ = (no. of reads * bin size)/genome size, where λ, the mean number of reads per bin, is obtained using negative binomial distribution [13]. For this, a configuration file providing information regarding the total length of the chromosome, expected percentage of gain and loss (default: 0.05), FDR (0.01) and dispersion values (default: 3) needs to be provided by the user. For the chosen bin size, the function ***prepareData*** will construct the ‘coordinate file’ of non-overlapping bins required as input for the pre-treatment step.

### 2. Data pre-treatment and filtering

NGS techniques generate short sequence reads (36–150 bp), suffer from non-uniform coverage since sequencing is biased with respect to DNA content, i.e., some regions amplify more efficiently than others, introduce platform-specific sequencing artefacts and high base-call error rates during DNA fragmentation (sonication), library preparation or sequencing steps. This greatly affects the quality of alignment, especially in low complexity regions of the genome. Systemic biases such as GC-bias and mappability bias are two leading causes of non-uniform coverage of reads. If not corrected, the read-depth signal computed from raw aligned reads may not represent the true copy number along the length of the genome. Below we discuss the approaches used in *i*CopyDAV to handle these biases.

#### GC-bias correction

A common observation across all NGS platforms is the under-representation of DNA fragments from low GC or high GC regions of the genome resulting in a bias in the estimation of RD signals in these regions. In *i*CopyDAV, two approaches are provided for GC-bias correction: (i) Local Polynomial Regression fitting (Loess) algorithm [44] and (ii) Median approach by Yoon et al [17]. The user also has an option to not opt for GC bias correction. In Loess regression approach, reads in bins with similar GC content within an interval of 0.001 are clubbed together. Loess correction is applied to each bin, computed based on the difference in the average read depth value of the bin and median read depth value for the whole genome (span = 0.75 (default), controls degree of smoothness). The median read depth is computed in such a way that GC adjustments do not have any effect on the overall median of the data. In the Median approach for GC bias correction, the corrected read depth of each bin *i* is given by the expression,

$${\tilde{r}}_{i}=r_{i}\frac{m}{m_{GC}}$$ (1)

where *r_i_* is the read count of the *i*^*th*^ bin, *m_GC_* is the median read count of all bins having the same G+C content as the *i*^*th*^ bin, and *m* is the overall median read count of all the bins. The R functions *loessGCCorrection* [13] and *gcCorrectionYoon* [17] perform GC bias correction by Loess-regression and Median-based approaches, respectively. GC profile plot generated helps the user to confirm whether the read depth in low and high GC content regions are properly correctly (shown in S1 Fig). Both these approaches require a file containing bin-wise GC content scores which can be obtained from UCSC genome browser [45] for human reference genome (available with 5 bp as bin size). Using this one can compute the GC scores file (*gcfile*) for any specific bin size and genome assembly using the function ***prepareData*** in *i*CopyDAV (the GC score files for bin size = 100 bp are available for hg18 and hg 19 assemblies at the website).

#### Handling mappability bias

Mappability of a region in the reference genome is defined as the probability that a read originating from it is unambiguously mapped back to it. It depends on the length of the reads, the number of mismatches allowed during the mapping step and the reference assembly used. Reads that cannot be uniquely mapped to the genome are considered unmappable, and the genomic positions from where they originate are called as unmappable regions. Bin-wise mappability scores (0–1) can be computed along the chromosome/genome. Regions of low mappability score (< 0.5) correspond to repetitive or low-complexity regions in the genome. For handling mappability bias in *i*CopyDAV, the user may define the mappability cut-off score, Mth (default = 0.5), with ‘0’ corresponding to no mappability bias correction. Then bins with mappability scores > Mth are only considered for subsequent steps in the pipeline. Mappability score tracks can be obtained from UCSC genome browser and computed for any bin size, similar to the construction of GC score file discussed above or by using gem library *[46]*. Using function ***prepareData*** one can generate mappability file (*mapfile*) for any desired bin size by using the mappability score file for 100 bp bin size (available at the website).

In *i*CopyDAV, the module ***pretreatment*** calculates raw RD values for the bin coordinate file, corrects the raw RD values of each bin appropriately using one of the above GC bias correction methods and filters low mappable reads. For this bin coordinate file (***z***), mappability scores (***mapfile***) and GC content scores (***gcfile***) are required.

### 3. Segmentation

The pre-treated data is now ready for the segmentation step to identify contiguous regions having same copy number (i.e., similar read count values). In *i*CopyDAV, we provide two segmentation approaches: Total Variation Minimization (TVM) algorithm based on agglomerative (or bottom-up) approach, wherein the adjacent bins having similar RD values are merged into larger segments, and Circular Binary Segmentation (CBS) algorithm based on divisive (or top-down) approach, in which the genomic regions are divided into segments such that the bins in a segment have similar read depth (RD) values. In both these approaches, the segmentation process is viewed as a change point-detection problem, with change-points as the genomic locations where read depth changes. The two segmentation approaches are briefly discussed below.

#### Total variation minimization (TVM) approach

We have implemented the TVM approach proposed by Duan et al [27] in C++. In this approach, the read depth profile is assumed to be a piecewise constant function of plateaus and basins modelling duplication and deletion events respectively along the length of the chromosome. At every step, two adjacent bins with minimum deviation in their RD signals are merged into a single segment with average RD value of the two merged segments and the process is continued iteratively. At every merging step, a total variation penalized least squares (*v*) is used to fit the RD profile using the equation:

$$v=\min\left\{\frac{1}{2}\sum_{i=1}^{n}{\left(y_{i}‑x_{i}\right)}^{2}+\lambda \sum_{i=1}^{n‑1}\phi \left(x_{i+1}‑x_{i}\right)\right\}$$ (2)

where the first term on the right is the fitting error between original signal *y*_*i*_ and the recovered smooth signal *x*_*i*_ in the *i*^*th*^ bin; the second term is the total variation penalty imposed when a change-point is detected between *x*_*i*_ and *x*_i+1_ and λ is the penalty parameter which controls the trade-off between fitting error and penalty caused by the change-points [27]. Schwarz information criterion (SIC) has been implemented to compute the optimal value of λ [47], given by

$$\mathrm{SIC}\left(\lambda_{k}\right)=m\;ln\left(n\right)+\frac{\sum_{i=1}^{n}{\left(y_{i}‑\tilde{x_{i}}\right)}^{2}}{\sigma^{2}}$$ (3)

where mean of *y*_*i*_ in a segment is used as the amplitude *x*_*i*_ i.e., for a given segment spanning *i* to *i*+*l* region, ${\tilde{x}}_{i}={\tilde{x}}_{i+1}=...={\tilde{x}}_{i+l}=\frac{\sum_{i}^{i+l}y_{i}}{l+1}$. Here *m* is the number of segments, σ^2^ variance of noise, and optimal λ is achieved at:

$$\hat{\lambda }=\arg\;\min_{k}\;SIC(\lambda_{k})$$ (4)

Once $\hat{\lambda }$ is known, the optimal smoothed signal of *y*_*i*_ is ${\tilde{x}}_{i}({\hat{\lambda }}_{k})$ and a CNV is identified as a segment with change-points at *i* and *i*+*l* and having amplitude below or above the predefined cut-off values.

#### Circular binary segmentation (CBS) approach

CBS is a recursive algorithm that partitions the bins *x*_*1*_, *x*_*2*_*…x*_*n*_ into a set of segments such that the read depth values in each segment are similar. The basic approach is as follows. In each step, it identifies an interval *i…j* such that the read depth values *x*_*i*_,*…*,*x*_*j*_ are all similar and significantly different from the rest of the regions, *x*_*1*_,*…*,*x*_*i-1*_ and *x*_*j+1*_,..,*x*_*n*_. If no such interval is identified, then the whole region *x*_*1*_,*…*,*x*_*n*_ is identified as a segment with no change point detected, else three intervals *x*_*i*_,*…*,*x*_*j*_, *x*_*1*_,*…*,*x*_*i-1*_ and *x*_*j+1*_,..,*x*_*n*_ are obtained (considering the genome with periodic boundary conditions (circular) effectively results in two segments, *x*_*i*_,*…*,*x*_*j*_ and *x*_*j+1*_,..,*x*_*i-1*_, with the larger fragment having average copy number 2). To determine if the segment *x*_*i*_,*…*,*x*_*j*_ is significantly different from the other regions, the null hypothesis of no difference is assumed and t-statistic is computed for each interval *i…j*, defined as

$$T_{i,j}=\frac{\mu_{i..j}‑{\bar{\mu }}_{i..j}}{\sqrt{\frac{1}{n_{i..j}}+\frac{1}{n‑n_{i..j}}}}$$ (5)

where *n_i..j_* (= *j-i+1*) is number of bins in the interval *i…j*, *n-n_i..j_* is the total number of bins in *x*_*1*_,*…*,*x*_*i-1*_ and *x*_*j+1*_,*…x*_*n*_, $\mu_{i..j}=\frac{x_{i}+\ldots +x_{j}}{n_{i..j}}$ is the mean read depth in the interval *i…j* and ${\bar{\mu }}_{i..j}=\frac{\left(x_{1}+\ldots +x_{i‑1})+(x_{j+1}+\ldots +x_{n}\right)}{n‑n_{i..j}}$ is the mean read depth for the remaining regions *x*_*1*_,*…*,*x*_*i-1*_ and *x*_*j+1*_,..,*x*_*n*_. This procedure is applied recursively to every interval *i…j* and the interval that maximizes the t-statistic, i.e., *T*_*max*_ *= max*_*1≤i<j≤n*_ *|T*_*i*,*j*_*|* is greater than a threshold (determined using a permutation test), the null hypothesis of no difference is rejected and the interval *x*_*i*_,*…x*_*j*_ is identified as a copy variant region. The Bioconductor package, DNAcopy [48], is incorporated for circular binary segmentation in the pipeline.

The segmentation step being computationally intensive, TVM is parallelized using OpenMPI *[49]*, while for CBS, parallel implementation of the ‘segment’ function, ParDNAcopy [50] is considered. This is achieved by dividing the reference sequence into ‘*p*’ equal parts (default *p* = 32) with an overlap of 20 bins/windows between adjacent parts (to handle copy number variant regions at the boundaries). The parameter *p* is an optional parameter and can be set according to available CPU cores. The segmentation is applied independently and in parallel on each of the *p* parts and, after the segmentation, the parts are merged back in proper order to identify segments having similar RD signal. Thus, by utilizing multi-core architecture, *i*CopyDAV is much faster (~ 8-fold) than most other CNV detection programs. It has been shown that CBS approach is suited for identifying large CNVs [13], while the efficacy of TVM approach is shown in the detection of small CNVs [27]. Thus, by combining the results of the two approaches, one would be able to detect wider range of CNVs in *i*CopyDAV.

Function ***runSegmentation*** with parameter **-*t*** for TVM and **-*d*** for CBS generates a coordinate file containing both normal and variant regions along with their corresponding RD values.

### 4. Variant calling

In this step, the segmented regions with deviant copy number are identified and reported with their start/end coordinates, type of event (duplication or deletion) and absolute copy number. First, the average RD value for the whole chromosome is computed by using a simple iterative convergence algorithm to ensure that the RD value of the variant regions is ignored while computing the mean. This is achieved by iteratively computing the average RD value of the segments, ignoring those with very high and very low RD values at every step. The iteration stops until the mean RD across the chromosome converges. The mean RD value of the chromosome is then used to define the upper and lower thresholds for identifying the deviant regions (allowing 5% error). For its simplicity and effectiveness, empirical thresholds given below are used to identify the variant regions and their absolute copy number:

UpperThreshold(UT)=1.45×(MeanRDvalue)

LowerThreshold(LT)=0.55×(MeanRDvalue)

$$\mathrm{Absolute}\;\mathrm{Copy}\;\mathrm{Number}\;\mathrm{of}\;a\;\mathrm{Region}=\left(\frac{\mathrm{RD}\;\mathrm{value}\;\mathrm{of}\;\mathrm{the}\;\mathrm{Region}}{\mathrm{Mean}\;\mathrm{RD}\;\mathrm{value}\;\mathrm{of}\;\mathrm{Chromosome}}\right)\times 2$$ (6)

Regions with RD value > upper threshold (UT) are reported as duplications and those with < lower threshold (LT) as deletions. To handle inaccurate splitting of a variant region due to the hard threshold used in the variant calling step, two adjacent variant regions of size *x* bp and *y* bp separated by a small non-variant region of size *n* bp are merged in the post-processing step if they are of the same event type (gain/loss), have similar copy number, and satisfy the following condition [51]:

$$\frac{n}{\left(x+y+n\right)}\leq 0.2$$ (7)

In *i*CopyDAV, variant calling is done on the files generated in the segmentation step by the using function ***callCNV***. The variant regions are reported in a tab separated format (BED) containing chromosome number, start and stop positions of CNVs, event type (duplication: 1, deletion: 0) and absolute copy number.

### 5. Annotation and visualization

The annotation module in *i*CopyDAV identifies various structural and functional elements in the vicinity of (or within) the predicted CNVs and are summarized in Table 3. Using the function ***annotate***, the user can obtain four categories of annotations mapped to the CNVs, *viz*., (1) functional elements, (2) clinical associations, (3) known CNVs reported in the Database of Genomic Variants (DGV), and (4) other structural variations (e.g., Segmental duplications, repeats) and genomic regions such as telomeres, centromeres. Based on these annotations, the CNVs are assigned priority: high, medium or low.

**Table 3:** Structural and functional annotations provided by the *annotate* module in *i*CopyDAV.

Annotation	Feature	Method/source	Database	Overlap Criteria	Priority class
Functional	Protein-coding gene	Refseq [52]	UCSC genome browser	>1 bp	Medium
	Gene elements (exon, UTR, start/stop)	Gencode [53]	Gencode	>1 bp	Medium
	Enhancers	Vista [54]	UCSC genome browser	>1 bp	Medium
	lincRNA	Cufflinks [55]	UCSC genome browser	>1 bp	Medium
	miRNA target sites	TargetScanS [56]	UCSC genome browser	>1 bp	Medium
Clinical	Pathogenicity & disease	ClinVar [57]	ClinVar	50% with CNV	High
	OMIM disease ID	OMIM [58]	OMIM	50% with CNV	High
	Haploinsufficiency index	DECIPHER [59]	DECIPHER	>1 bp	High
	Genic intolerance of rare CNV	ExAC [60]	ExAC	>1 bp	High
Known	DGV Id	DGV [61]	DGV	50% with CNV	Low
Structural	Heterochromatin and telomeric regions	Cytoband [45]	UCSC genome browser	50% with CNV	NA
	Segmental duplication	Whole-genome assembly comparison method (WGAC) [62]	UCSC genome browser	50% with CNV	NA
	Tandem repeats	TRFinder [63]	UCSC genome browser	50% with CNV	NA
	Interspersed repeats	RepeatMasker [64]	UCSC genome browser	50% with CNV	NA

OMIM: Online Mendelian Inheritance in Man, DECIPHER: Database of Genomic Variation and Phenotype in Humans using Ensembl Resources, ExAC: Exome Aggregation Consortium, DGV: Database of Genomic Variants

It has been observed that large CNVs may affect multiple protein-coding genes or truncate a functional gene which may lead to clinical conditions via mechanisms of gene dosage imbalance (haploinsufficiency and triplosensitivity), gene disruption or gene fusion events, including non-coding variant effects [65]. Long non-coding RNAs (lncRNAs) and regulatory elements including enhancers and miRNA target regions spanning CNVs have been shown to have a direct/indirect impact on gene regulation [66]. From literature there is evidence to support CNVs in the non-coding regions can also be pathogenic by a position effect mechanism [65]. In *i*CopyDAV, we provide annotations of protein-coding and non-coding genes and other functional elements encompassing CNVs. For clinical significance of the predicted CNVs, information from four resources is incorporated. Disease association is obtained from ClinVar [51] and OMIM [52] databases, and pathogenicity information from ClinVar, while haploinsufficiency and genic intolerance information is obtained from DECIPHER [53] and ExAC [54], respectively. When a single functional copy of a gene is insufficient to maintain normal function, it is said to be haploinsufficient [59]. Based on the predicted probability, higher ranks (e.g. 0–10%) indicate a gene is more likely to exhibit haploinsufficiency, while the genic intolerance scores obtained from ExAC, indicate whether a genic CNV can be identified as a common functional genetic variation relative to expected genome-wide variation. The predicted CNVs are also searched against the Database of Genomic Variants (DGV) [55] to identify known, population-specific CNVs. This would help in filtering out common population-specific CNVs and enable prioritization of phenotype-specific rare CNVs. The structural genomic features in the vicinity of the predicted CNVs, e.g., segmental duplications, tandem repeats and interspersed repeats, and their localization (e.g., heterochromatin, telomeric regions) are extracted from UCSC Genome Browser. These structural annotations provide insight into the mechanism of CNV formation. For example, CNVs in the proximity of segmental duplications, interspersed repeats and tandem repeats are likely to be formed as a result of non-allelic homologous recombination (NAHR). Based on the extent of overlap one can distinguish between true CNVs from other structural variations.

The above annotations are collated and CNVs are assigned a priority based on their probable functional impact. CNVs overlapping with clinically significant variants are given ‘High’ priority, while overlap with DGV CNVs is given ‘low’ priority. Novel CNVs which span any of the functional elements are assigned ‘medium’ priority, while no such categorization is provided for CNVs with structural annotations.

The visualization module in *i*CopyDAV, ***plot***, allows the user to plot the distribution of CNVs along the chromosome or a region of interest (user defined coordinates), which can be downloaded in ‘.png’ format. The distribution of CNVs is also depicted as an ideogram.

## Results

The performance of *i*CopyDAV is evaluated on simulated sequence data and real chromosome data. In simulation experiment, the performance of the two segmentation algorithms in *i*CopyDAV is assessed in their ability to identify CNVs of varying size, breakpoint accuracy and copy event (gain/loss) as a function of sequencing coverage (5× - 50×). The choice of data pre-treatment and segmentation methods on variant calling are also discussed. For the analysis on real data, Chr1 of NA12878 Caucasian sample (CEPH) is considered at two sequencing depths (6× and 35×). The predicted CNVs in each are validated against 6 different studies reported in Database of Genomic Variants (DGV) for NA12878 sample. Finally, the functional analysis of predicted CNVs is discussed.

### 1. Simulation experiment

To simulate CNV detection in a real genome with all its complexities and compositional biases, a contiguous segment of length 40Mb from position 75,671,302 to 115,671,302 of human chromosome 4 (hg18 assembly) is taken, referred as 'reference'. In a copy of this 'reference' sequence, 24 CNVs of lengths varying from 100–5000 bases, comprising both duplications and deletions (copy number 1, 3 and 4) are inserted (summarized in S1 Table). This is then concatenated with the 'reference' to simulate a diploid genomic sequence, referred as 'simulated sequence' hereafter. Single end reads of length 50 bp are generated from this simulated sequence using ART simulator [67] at six different sequencing depths, 5×– 50×. Reads are aligned to the ‘reference’ using Bowtie2 aligner [68] to obtain alignment files in BAM format. For each sequencing depth, this exercise is repeated 50 times and the predictions are averaged over these repetitions.

#### Analysis of simulated data

For the simulated data, GC bias correction is carried out using Median-based approach and regions of low complexity regions are removed using mappability threshold, Mth = 0.5 (default). A small bin size of 50 bp is considered. Performance of the two segmentation approaches is evaluated using F-score, defined as the harmonic mean of precision and recall (sensitivity):

$$F=\frac{2\times \mathrm{recall}\times \mathrm{precision}}{\left(\mathrm{recall}+\mathrm{precision}\right)}$$ (8)

where, recall is defined as the proportion of true CNV predictions (TP) to the total number of CNVs inserted (P):

$$\mathrm{Recall}=\frac{\mathrm{TP}}{P}=\frac{\mathrm{Number}\;\mathrm{of}\;\mathrm{CNVs}\;\mathrm{correctly}\;\mathrm{predicted}}{\mathrm{Total}\;\mathrm{number}\;\mathrm{of}\;\mathrm{actual}\;\mathrm{CNVs}}$$ (9)

and precision is defined as the ratio of correctly predicted CNVs (TP) to the total number of CNV predictions (TP + FP):

$$\mathrm{Precision}=\frac{\mathrm{TP}}{\mathrm{TP}+\mathrm{FP}}=\frac{\mathrm{Number}\;\mathrm{of}\;\mathrm{CNVs}\;\mathrm{correctly}\;\mathrm{predicted}}{\mathrm{Total}\;\mathrm{number}\;\mathrm{of}\;\mathrm{CNVs}\;\mathrm{predicted}}$$ (10)

The criterion for ‘true positive’ considered here is reciprocal overlap of 50% or more between the predicted and actual CNVs. Performance of *i*CopyDAV is assessed at four levels in the simulated experiment: (i) size of CNVs, (ii) breakpoint accuracy, and (iii) event type (duplication/deletion) as a function of sequencing depth, and (iv) for comparison with three other DoC based approaches.

#### Performance of CNVs on simulated data

**Fig 2:**
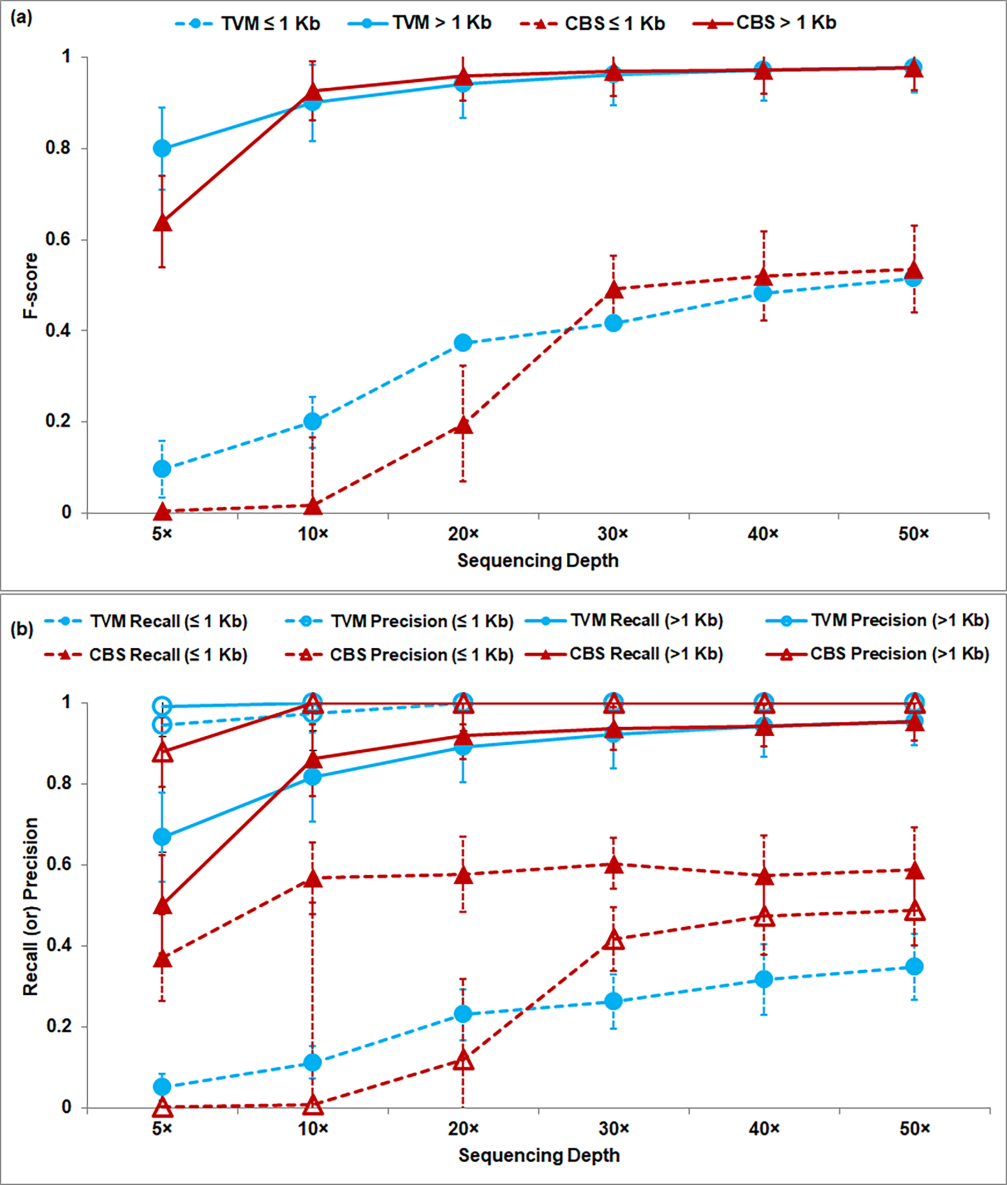
(a) F-score plots, and (b) Recall & Precision plots as a function of sequencing depth. In (b) Recall and precision values are depicted by ‘closed’ and ‘open’ symbols respectively. Performance of TVM (‘circle’) and CBS (‘triangle’) is shown for the two CNV sets: small (≤ 1Kb) shown as dashed lines and large (> 1Kb) as solid lines. Error bars represent standard deviation in each group. Bin size = 50 bp.

**Size of CNVs:** To analyze the efficacy of *i*CopyDAV in the detection of ‘small’ and ‘large’ CNVs, the inserted CNVs are split into two groups: small (0.1–1 Kb) and large (> 1–5 Kb). CNVs larger than 5Kb are not considered here as most algorithms perform well in the detection of large CNVs. Homozygous deletions and copy gain > 4 are also not considered as these are easy to detect. Figs 2(A) and 2(B) respectively depict the F-scores and precision-recall curves as a function of sequencing depth for the two CNV sets, the ‘dashed’ curves corresponding to the performance of ≤ 1 Kb CNV set, and the solid curves for > 1 Kb CNVs. It is observed from Fig 2(A) that the prediction accuracy for CNVs > 1Kb is quite good, greater than 90% for sequencing coverage ≥10×, while that for CNVs ≤ 1 Kb it is ~50% for sequencing depth (≥ 30×) with both the segmentation approaches. This clearly indicates that larger CNVs can be easily detected even in low coverage data by any segmentation approach, but higher sequencing depth is required for the detection of smaller CNVs (especially for single copy gain/loss events). From Fig 2(A) it is observed that TVM approach performs marginally better than CBS approach in the detection of small CNVs (≤ 1 Kb) in low coverage data (< 30×).

High recall values indicate that most of the true CNVs are correctly detected, while high precision values indicate fewer false positives. In general, a balance between the two is desired for any algorithm. It is observed from Fig 2(B) that both recall and precision are high and comparable for TVM and CBS approaches in the detection of CNVs of size > 1Kb and approach ‘1’ with increase in sequencing coverage (≥ 20×). However, for detecting CNVs of size ≤1 Kb, we observe that CBS has a better recall while TVM gives better precision (~ 1 even at 5×), with the overall performance being marginally better for TVM approach (Fig 2(A)). This suggests that for the detection of small CNVs (≤1Kb), a combination of the two approaches may be useful.

**Fig 3:**
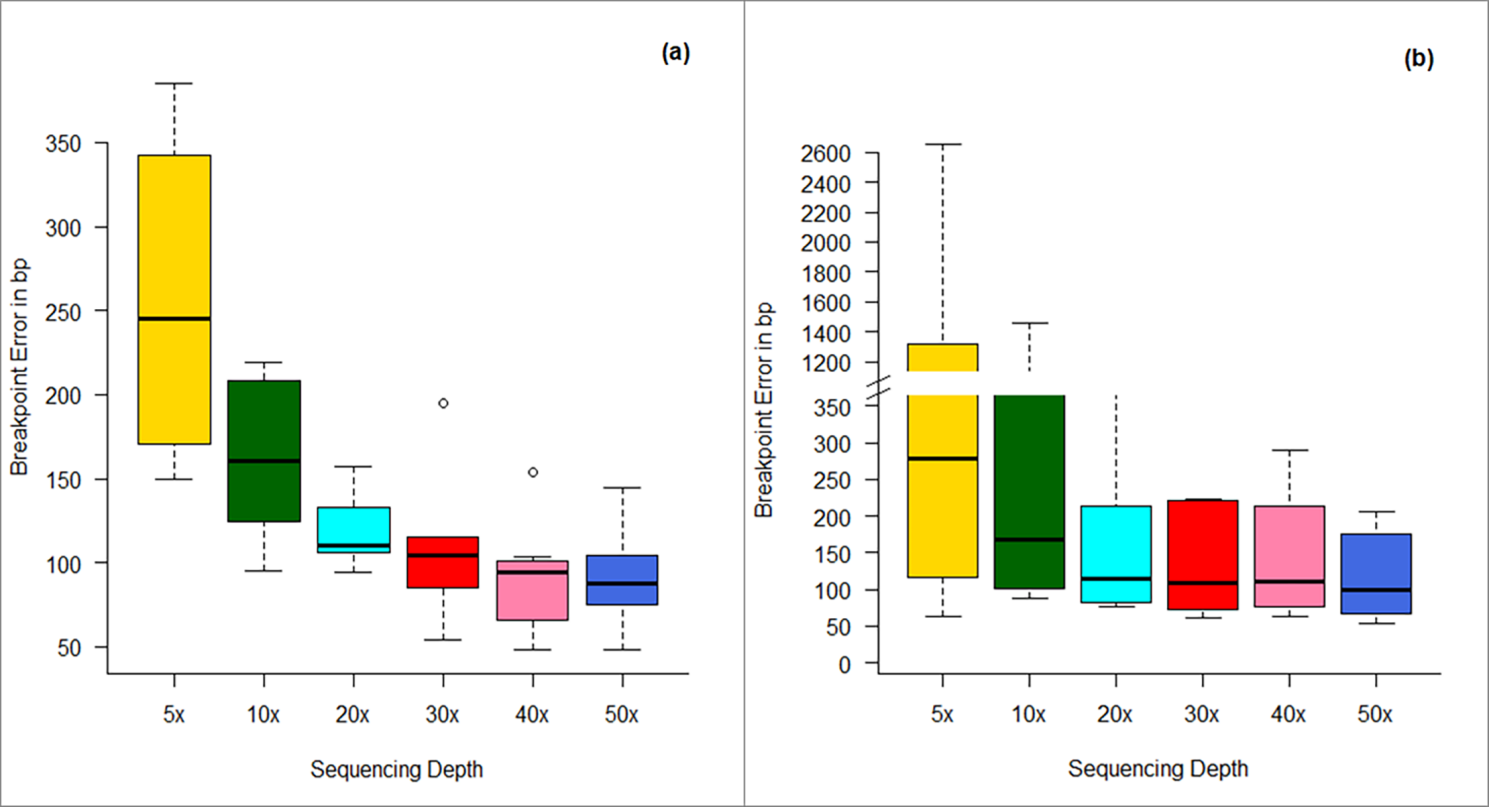
Box plots representing breakpoint error in CNV detection as a function of sequencing coverage using (a) TVM and (b) CBS segmentation approaches in simulated data. Bin size = 50 bp.

**Breakpoint accuracy:** The error in accurate detection of the start/end boundaries of predicted CNVs is defined as the breakpoint error. The breakpoint error in the prediction of 24 CNVs in our simulation experiment as a function of sequencing coverage is summarized as boxplots in Fig 3. As expected, the average breakpoint error and the variation in the error decrease with increase in the sequencing depth. This may probably be due to reliability in the alignment of reads and better resolution of the segmentation approaches at higher sequencing coverage. For 30× and higher sequencing coverage, the median breakpoint error is about ~ 2 bin sizes for both the approaches, but with higher variation observed in the case of CBS approach ~ 109 (62–490) compared to the case of TVM approach, ~105 (54–195) at 30× sequence coverage. Very large variation in the breakpoint error is observed in the case of CBS approach, though the Median value is comparable in the two cases, especially for low coverage data (≤ 30×). This probably could be due to the requirement of larger bin sizes for optimal performance of CBS segmentation approach in low sequence coverage data. Since TVM approach performs well with smaller bin sizes, a better resolution of the breakpoints is observed in this case (≤ 30×).

**Fig 4:**
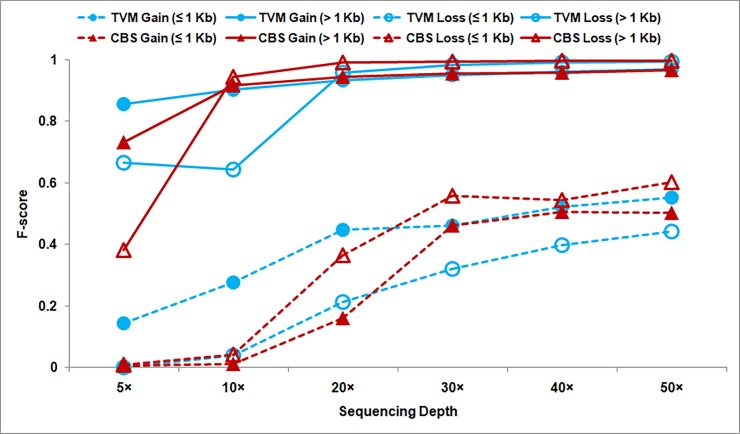
Performance of TVM (‘circle’) and CBS (‘triangle’) approaches in predicting copy gain (‘closed’ symbol) and copy loss (‘open’ symbol) events in simulated data is shown. Dashed line corresponds to detection of small CNVs (≤ 1 Kb) and solid line for large CNVs (> 1 Kb). Bin size = 50 bp.

**Type of CNVs:** To see if there is any bias in the detection of copy gain or copy loss events, the F-scores are computed for the two segmentation algorithms and depicted in Fig 4 for each case. No difference is observed in the detection of large copy gain and copy loss events (> 1 Kb), and F-scores are observed to approach ~ 1 for sequencing coverage ≥ 20× for both the segmentation approaches. For sequencing coverage < 20×, a slight bias in the detection of copy gain events is observed suggesting that it is easier to detect duplications than deletions in low sequence coverage data. But this bias is likely to be due to copy number 4 events which are easier to detect compared to single copy gain and copy loss events (3 and 1). In the detection of small CNVs (≤ 1 Kb), TVM approach is much better in detecting copy gain events while the performance of both the approaches is very poor in detecting copy loss events (when sequencing depth is ≤ 20×).

**Effect of bin size:** The bin size is an important parameter and affects CNV detection by DoC-based approaches. We observe that optimal performance for TVM approach is observed on using small bin size (~50 bases), while for CBS approach larger bin sizes are required (see S2 Fig), when sequence coverage is low (≤ 20×). For high sequence coverage data, no such dependence on bin size is observed and a small bin size is desirable for breakpoint accuracy. Since small bin size result in smaller breakpoint error, TVM approach is best suitable when sequence coverage is low as observed above in computing breakpoint accuracy.

**Fig 5:**
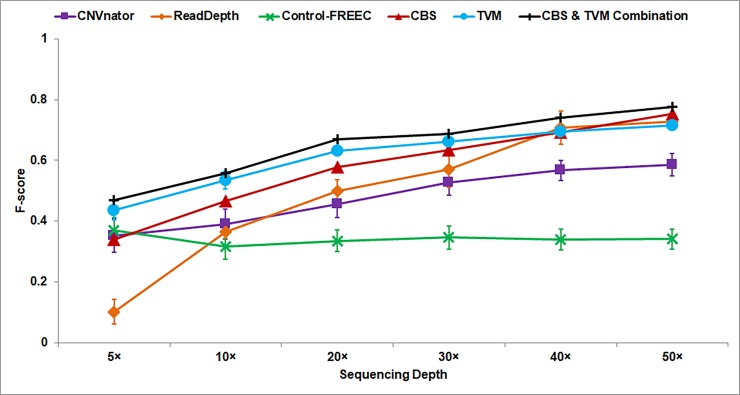
Performance (F-score) of *i*CopyDAV with three other DoC-based tools (using default parameters for data pre-treatment and segmentation approaches) shown as a function of sequencing coverage. Error bars represent standard deviation in each group.

**Comparison with other DoC-based Methods:** The performance of *i*CopyDAV on simulated data is compared with three DoC-based tools, *viz*. CNVnator [18], ReadDepth [13] and Control-FREEC [7], using their default parameters for data pre-treatment and segmentation approaches. Bin size is set to 50 bp in Control-FREEC, CNVnator and TVM segmentation algorithm in *i*CopyDAV; while for ReadDepth and CBS approach in *i*CopyDAV, optimal bin size is computed using negative binomial distribution for each sequencing depth: 1800 bp (5×), 900 bp (10×), 500 bp (20×), 400 bp (30×), 300 bp (40×) and 200 bp (50×). We expect the difference, if any, in the performance of these tools to arise as a consequence of different pre-treatment and segmentation approaches. For instance, CNVnator uses Median approach for GC bias correction and Mean-shift algorithm for segmentation; Control-FREEC uses Polynomial fitting for GC bias correction and Lasso-regression for segmentation, while ReadDepth uses Loess regression for GC bias correction and circular binary segmentation (CBS). For this comparative analysis, in *i*CopyDAV, Median approach is used for GC bias correction for both TVM and CBS approaches. Different approaches are used for handling mappability bias in these tools; low mappable regions are filtered by setting a mappability threshold (Mth). In Control-FREEC (Mth = 0.85), *i*CopyDAV (Mth = 0.5), and ReadDepth (Mth = 0.75 and additionally normalized by Mth), while multi-reads spanning low mappable regions are randomly assigned in CNVnator. All the tools consider raw read depth values for segmentation, except ReadDepth which uses log-transformed values. From Fig 5 we observe that both TVM (cyan circles) and CBS (red triangles) approaches in the *i*CopyDAV pipeline perform better than the other three DoC-based approaches and the combined predictions from TVM+CBS (black pluses) show further improvement in prediction. For the combined predictions of TVM and CBS, we consider the Union of the set of CNVs detected by the two segmentation approaches, that is, CNVs detected by either of the two approaches. For the CNVs detected by both the approaches, the farthest start/stop boundaries are considered. It may be noted that though both ReadDepth and CBS in *i*CopyDAV uses the same segmentation approach, the performance of CBS is better than ReadDepth for sequencing coverage < 40× due to the difference in data pre-treatment approaches in two cases.

From the comparison of recall and precision values of CBS and TVM in *i*CopyDAV with the three DoC-based tools, we observe higher recall with both TVM and CBS approaches for the detection of CNVs ≤ 1 Kb, while for the detection of CNVs > 1 Kb all the DoC-based methods showed recall values approaching ‘1’ with increase in sequencing coverage (S3(A) Fig). The precision ~ 1 with both CBS and TVM in the detection of small and large CNVs (even at 5× sequencing coverage for TVM), while precision values (combined of small and large CNVs) of ReadDepth, CNVnator, and Control-FREEC are ~ 0.71, ~ 0.51 and ~ 0.26, respectively, at 30× coverage (S4(B) Fig). Also, it is observed that in case of ReadDepth and Control-FREEC, a reduction in precision is observed with increase in sequencing depth due to increase in the number of false positives. This clearly indicates the importance of appropriate normalization of read depth signals. We observe that the performance of *i*CopyDAV is further improved by combining the output of the two segmentation approaches (S5 Fig), especially for small CNVs (≤ 1 Kb).

### 2. Real data

To test the efficacy of *i*CopyDAV on real data, we consider Chromosome 1 of NA12878 Caucasian (CEPH) sample which is most extensively studied and well-annotated for structural variations by various studies. To study the effect of sequence coverage on CNV prediction, data for this sample is considered at two different sequencing depths: low (6×) and high (35×). For low sequence coverage data (6×) obtained from the 1000 Genome Project consortium (2015) [62] (sample ID: SRR622461), paired-end reads of size 100bp are aligned to Chromosome 1 (hg18 assembly) using BWA-MEM (with default parameters) to obtain the alignment file in BAM format. For high sequence coverage data (35×), alignment file available in BAM format is downloaded from the 1000 genome project. For this sample paired-end reads of size 36 bp have been mapped to hg18 assembly using MAQ aligner. Considering bin size of 300 bases, the function ***prepareData*** is used to construct the ‘coordinate file’ of non-overlapping bins and GC score files (*gcfile*) that are required as input for the pre-treatment step. Mappability score tracks are obtained from UCSC genome browser and mappability files (*mapfile*) are constructed for the two samples independently as the reads are of different lengths. Below we discuss our analysis for different combinations of data pre-treatment and segmentation approaches on the NA12878 sample at two sequencing depths, 6× and 35×.

#### a. Low sequence coverage data

**Fig 6:**
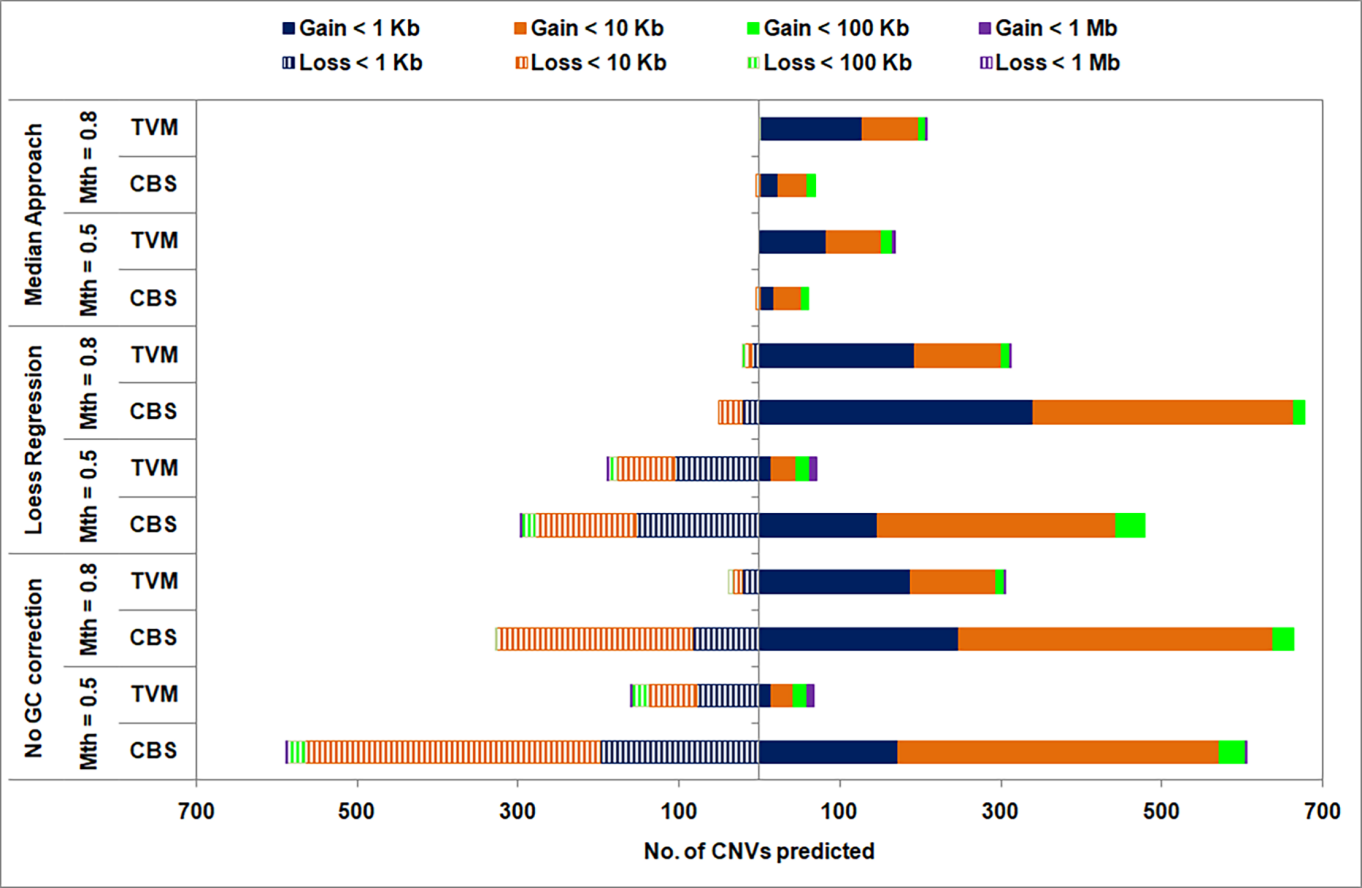
Size distribution of copy gain (solid) and copy loss (striped) events shown for various combinations of data pre-treatment and segmentation approaches in low sequence coverage data (6×) for Chr 1 of NA12878 sample. Bin size = 300 bp.

For various combinations of data pre-treatment and segmentation approaches in *i*CopyDAV, the number, type and size of CNVs predicted in low sequence coverage data are summarized in Fig 6. A large variation is detected in the type and size of CNVs. The number of CNVs predicted is very large using CBS approach in the case of no GC bias correction (for both high and low mappability cut-offs), clearly indicating the need for removing GC bias in this case. However, on using TVM segmentation approach, the number and type of CNVs predicted are comparable for Loess GC bias corrected and uncorrected data. The other major observation is that Median GC bias corrected data (with either TVM or CBS segmentation approach) resulted in very small number of CNVs, especially deletions, compared to other combinations. It is observed that for Loess GC bias corrected data, the number of deletion events are reduced on increasing mappability cut-off from Mth = 0.5 to 0.8. On the other hand, the duplications events are seen to increase on increasing mappability cut-off from Mth = 0.5 to 0.8 for all the cases. To test the reliability of predictions for various combinations of data pre-treatment and segmentation approaches, below we discuss recall and precision of the CNV calls by comparing with annotations in Database of Genomic Variations (DGV).

To access the reliability of various data pre-treatment and segmentation approaches in low sequence coverage data, variant calls are benchmarked against six studies reported in DGV for the sample NA12878 (summarized in S2 Table). Of these, four studies are based on SNP-array technique (Altshuler et al [69], Cooper et al [70], McCarroll et al [71] and Redon et al [72]), while the fifth study is a collection of CNVs predicted from four different techniques, *viz*., Fosmid mapping (Kidd et al [73]), array-CGH (Conrad et al [74]), SNP-array (McCarroll et al [71]) and whole shotgun sequencing (Mills et al [75]) and reported as ‘Gold standard’ in Mills et al [36]. The sixth study is based on sequence-based techniques (part of the 1000 Genome Project [76]) and comprises experimentally validated set of CNVs. For evaluating the performance, a predicted CNV is considered to be a true call (true positive) if ≥ 50% overlap is observed between prediction and annotation or vice versa. Due to large variation in the size of predicted and annotated CNVs, many-to-one (number of small adjacent CNV predictions mapping to a single large annotated CNV) and one-to-many mappings (a single large predicted CNV mapping to a number of small annotated CNVs) is typically observed. In such situations, to avoid over-estimation of recall, all adjacent predictions mapping to a single annotation are merged and counted as a ‘single’ CNV call while computing recall, and a single CNV prediction mapping to *m* annotated CNVs is counted as *m* CVN calls to avoid under-estimation while computing precision values. That is, recall is computed as the fraction of number of annotations (*l*) overlapping with the CNV predictions to the total number of annotations (*m*), i.e., recall = *l*/*m*, and precision is computed as the fraction of the number of true predictions (*x*) overlapping with the annotations to the total number of CNVs predicted (*y*), i.e., precision = *x*/*y*. Also, due to redundancy among the annotated CNV sets across the six studies, we assess our predictions independently on those studies.

**Fig 7:**
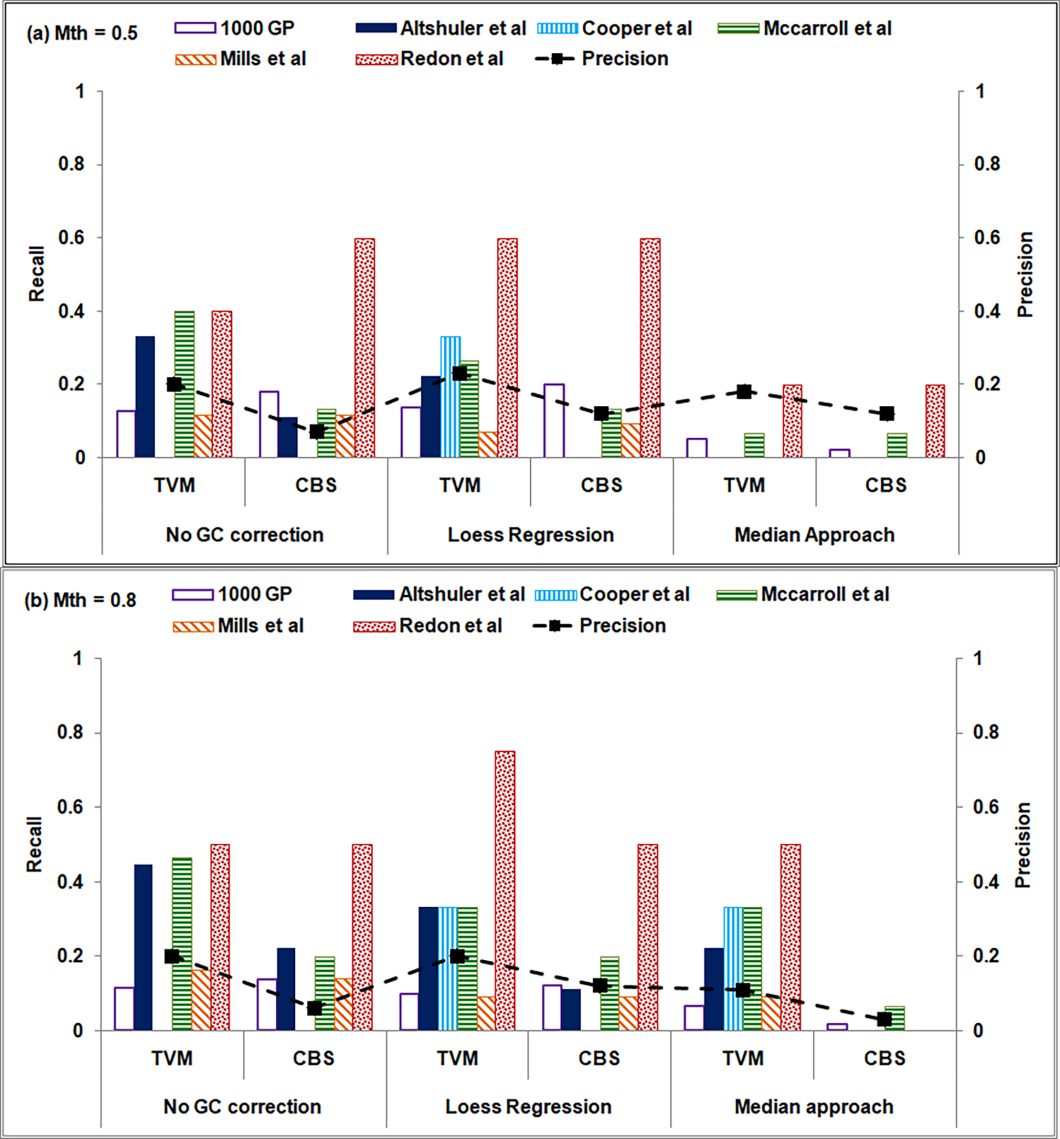
Performance of *i*CopyDAV is shown for low sequence coverage data (6×) of Chr 1 of NA12878 for mappability threshold values (a) 0.5 and (b) 0.8. Recall and precision values for various combinations of GC bias correction and segmentation algorithms are computed with respect to the six studies reported in DGV. Bin size = 300 bp.

Recall and precision values of CNV predictions for various GC bias correction and segmentation approaches are depicted in Fig 7(A) and 7(B) for two mappability cut-off values, Mth = 0.5 and 0.8, respectively. The recall values are plotted as bar graphs while the precision values are averaged across the six studies and depicted as a ‘black square’ (connected by a dashed line) for each combination of GC bias correction and segmentation approaches.

From Figs 6 and 7 we observe that the small number of CNV predictions observed in the case of Median + (TVM/CBS) combination resulted in low values of recall and precision for Mth = 0.5. However, on increasing Mth to 0.8, recall and precision improved in case of TVM segmentation approach. For Loess + CBS combination, though the number of CNV predictions is quite high, low recall and precision values observed suggest that majority of these are likely to be false positives. In the case of TVM approach, the recall and precision values for no GC bias correction case are comparable to GC bias corrected (Loess) data, indicating its robustness in CNV detection in raw, untreated data. Thus, Loess + TVM combination in *i*CopyDAV with Mth = 0.5 results in the best performance for low coverage data. We also observe that duplications are detected more accurately (46%) than deletions (12%) in this case, indicating that detection of deletion events is difficult in low coverage data. Similar observations were made in chromosomes 2 and 21 of NA12878 (results not shown).

#### b. High sequence coverage data

**Fig 8:**
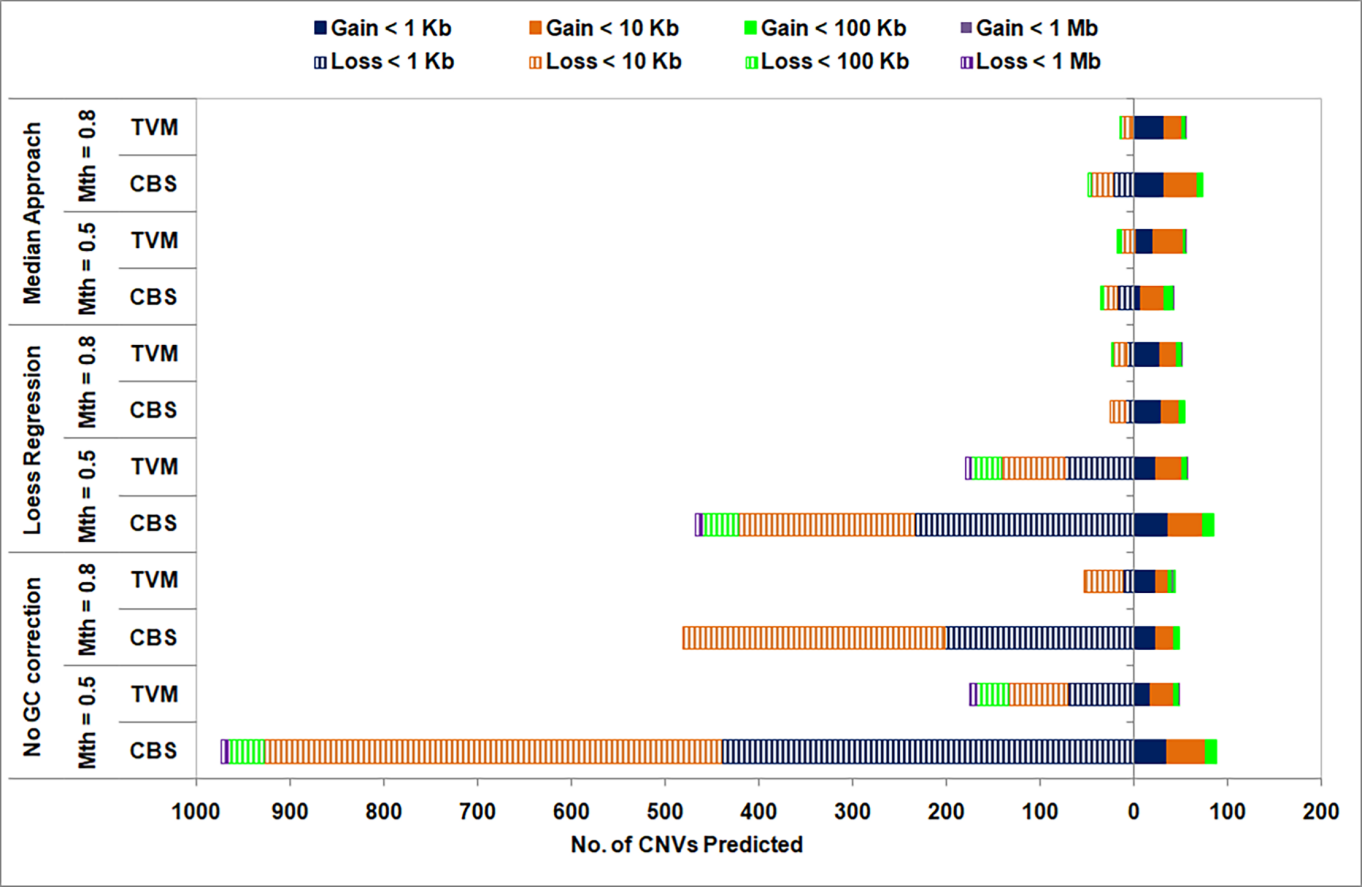
Size distribution of copy gain (solid) and copy loss (striped) events shown for various combinations of data pre-treatments and segmentation approaches in high sequence coverage data (35×) for Chr 1 of NA12878 sample. Bin size = 300 bp.

**Fig 9:**
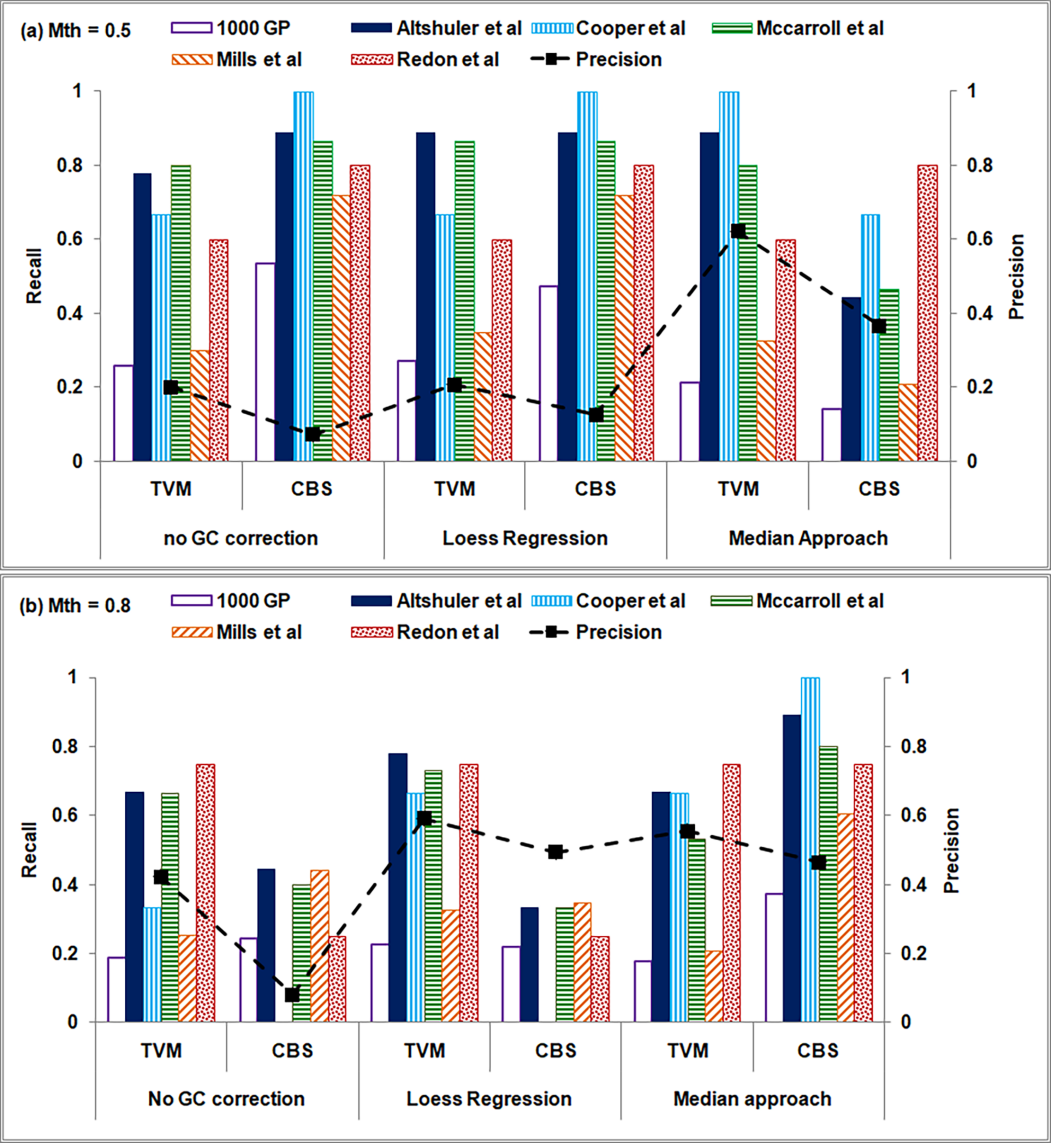
Performance of *i*CopyDAV is shown on high sequence coverage data (35×) of Chr 1 of NA12878 sample for mappability threshold values (a) Mth = 0.5 and (b) Mth = 0.8. Recall and precision values for various combinations of GC bias correction and segmentation algorithms are computed independently for the six studies reported in DGV. Bin size = 300 bp.

A similar analysis is carried out on high sequencing coverage data (35×) of Chr 1, NA12878 sample and the results are summarized in Figs 8 and 9. We again observe a large variation in the type and size of CNVs predicted for different combinations of data pre-treatment and segmentation approaches (Fig 8). This clearly suggests that one should judiciously choose the pre-treatment options along with the segmentation approaches. As in the case of low coverage data, large number of CNVs are predicted (mainly deletions) using CBS approach in the case of no GC bias correction, which are significantly reduced on removing GC bias (much more on using Median approach compared to Loess approach). This clearly indicates the need for GC bias correction when using CBS segmentation approach. However, using TVM segmentation approach, the number of CNV predictions are comparable for GC bias corrected (Loess/Median) and uncorrected data when mappability cut-off, Mth = 0.8. This suggests that TVM segmentation approach is able to handle GC bias and this correction step may be skipped when using higher mappability cut-off values. As in the case of low coverage data, in this case also the Median approach for GC bias correction resulted in fewer CNVs (mainly deletions) with both TVM and CBS approaches compared to other combinations. A clear reduction in the number of CNVs (mainly deletions) is observed on increasing the mappability cut-off from 0.5 to 0.8, except for Median of GC bias corrected data. Thus, a clear dependence of the segmentation results on data pre-treatment choices is observed. As before, we assess the reliability of the CNV calls for various combinations of data pre-treatment and segmentation approaches by comparing with six studies annotated in DGV for the NA12878 sample.

In Fig 9(A) and 9(B) recall and precision values are depicted for two mappability thresholds, Mth = 0.5 and 0.8, respectively, in the case of high sequence coverage data. As before, recall values are plotted as bar graphs for the six studies annotated in DGV and an average precision value across all the six studies is depicted as a black ‘square’ for various combinations of GC bias correction and segmentation approaches. As expected, higher recall and precision values are observed in this case (Fig 9) compared to low coverage data (Fig 7). From Fig 9(A) we observe that though high recall values are obtained for all combinations of data pre-treatment and segmentation approaches (except Median+CBS), when mappability cut-off, Mth = 0.5, the precision values are low (except Median+TVM), indicating a large number of false positives. The precision values are observed to improve for GC bias corrected data (Loess/Median) on increasing the mappability threshold to 0.8 as seen in Fig 9(B), and optimal performance is observed for Loess+TVM and Median+CBS combinations with a trade-off in recall and precision values. Thus, it is clear from this analysis that to reduce false positives, appropriate filtering of reads is required depending on the sequence coverage in DoC-based methods. Further, it may be noted that in the case of high sequence coverage data, Median approach for GC bias correction resulted in higher recall and precision values compared to Loess approach or uncorrected data, while it is other way round in the case of low sequence coverage data.

Here, we would like to mention that the six DGV studies considered for evaluating the performance of *i*CopyDAV are not truly gold standards and each of these methods have their own advantages and limitations in CNV detection. For example, SNP array-based techniques show high sensitivity (up to 1 bp resolution) in identifying CNVs compared to other array-based techniques such as array-Comparative Genomic Hybridization (arrayCGH), BAC and FOSMID. However, this method suffers from limited coverage of the genome with only 3 reported CNVs in Cooper et al to 15 in McCarroll et al (S3 Table), and thus misses out on many true CNVs. Further, the overlap among these four studies itself is quite low, just 2 CNVs. On individually analyzing the performance of *i*CopyDAV on the four SNP array-based studies reported in DGV (Altshuler et al [69], Cooper et al [70], McCarroll et al [71] and Redon et al [72]), we observe recall values ranging from 67% - 78% and precision from 13% - 70% with TVM approach for GC bias corrected data (Loess), and much higher recall values 75–100% but with very lower precision 11% - 30% on using Median+CBS approach (Mth = 0.8). Low precision values obtained for some of the SNP array-based studies is due to very small number of CNVs reported in these studies (3–15).

In Mills et al [36] study, 43 deletions are referred as ‘gold standard’ and have been collated from four studies using diverse CNV detection methods (Fosmid mapping, array-CGH, SNP-array and whole genome shotgun sequencing). High recall (~ 60%) and precision (~ 52%) values are observed with Median+CBS approach (Mth = 0.8) in detecting copy loss events. The precision improved to ~ 83% on using Loess+ TVM approach but with lower recall (~ 33%), suggesting that Median+CBS approach is better at detecting copy loss events. CNVs reported in 1000 Genome project (1000 GP) are collated from various NGS-based approaches [17,18,77–80] and experimentally verified by PCR (small CNVs) and arrayCGH methods (large CNVs). In *i*CopyDAV, Median+CBS combination (Mth = 0.8) resulted in best recall (37%) and precision (41%) values for 1000 GP CNVs, while Median+TVM combination (Mth = 0.8) resulted in higher precision (56%) but at the cost of low recall value (18%). A cross comparison of deletions reported in 1000 GP (validated set) with those reported in Mills et al (‘gold standard set’) and *i*CopyDAV predictions (Median+CBS, Mth = 0.8) using 50% overlap criteria was carried out. We observe that 32 of the CNV-deletions reported in Mills et al dataset had an overlap with 1000 GP CNVs, while 30 CNV-deletions in *i*CopyDAV overlapped with 1000 GP CNVs, indicating the reliability of the *i*CopyDAV predictions. We also observed that 33 (size: 700 bp– 431 Kb) of 1000 GP CNV-deletions are not identified by any of the six studies, including *i*CopyDAV. This disagreement of CNV predictions between different approaches clearly indicates the dependence of the approach used for CNV detection.

**Size and Type of CNVs:** The size of the CNVs reported in the six DGV studies range from 617 bp to 542 Kb, with median size 5600 bp. Hence, to see if there is any bias in the size of CNVs detected in *i*CopyDAV, we split the predictions into two sets: ‘small’ (≤ 5 Kb) and ‘large’ (> 5 Kb), and evaluate the performance of these two sets independently. Bias, if any, in the detection of the type of CNVs (copy gain or copy loss events) is also carried out. The F-score values for various size and type of CNVs detected in *i*CopyDAV are summarized in Table 4 for various combinations of data pre-treatment and segmentation approaches in the case of high coverage data. From the table it may be noted that Median+CBS approach shows optimal performance in the detection of both ‘small’ and ‘large’ CNVs, and also in the detection of ‘copy gain’ and ‘copy loss’ events. The Loess+TVM approach, on the other hand, performs equally well in the detection of ‘large’ CNVs and ‘copy gain’ events, while its performance is poor in the detection of ‘small’ CNVs. This again indicates that appropriate combination of pre-treatment and segmentation approaches need to be considered for optimal performance. On combining the results of the two segmentation approaches (TVM+CBS), we observe that the overall recall is higher with Median approach (0.51) while precision is higher on using Loess approach (0.58) for GC bias correction. No dependence on GC bias correction method (Loess/Median) is observed on the overall performance, which is marginally improved compared to the individual segmentation approaches. Thus, in *i*CopyDAV the user can merge results from various workflows to improve the predictive power and remove the dependence of pre-treatment methods. On considering the intersection (consensus) of CNVs obtained from Median + (TVM + CBS) and Mth = 0.8, the precision is significantly improved (~ 63%), but at the cost of low recall values (~26%). Similar observations were made on other chromosomes of NA12878 and in high coverage genome sequence data of NA12891 and NA12892 Caucasian samples (results not shown).

**Table 4:** Performance of various combinations of GC bias correction and segmentation approaches in the detection of CNVs of different size (small/large) and type (copy gain/loss) are summarized for high sequence coverage data (Mth = 0.8).

Method	F-score				Overall		
	Small (≤ 5Kb)	Large (> 5Kb)	Gain	Loss	Recall	Precision	F-score
Loess + TVM	0.26	0.59	0.34	0.47	0.34	0.59	0.43
Loess + CBS	0.39	0.17	0.25	0.40	0.26	0.49	0.34
Median + TVM	0.20	0.49	0.35	0.41	0.26	0.56	0.35
Median + CBS	0.42	0.57	0.33	0.58	0.50	0.46	0.48
Median + (TVM + CBS)	0.44	0.60	0.33	0.59	0.51	0.47	0.49
Loess + (TVM + CBS)	0.43	0.57	0.38	0.59	0.43	0.58	0.49

**Comparison with other Depth-of-Coverage based Methods:** To evaluate the performance of *i*CopyDAV with other CNV detection tools, the combined set of CNVs obtained using Median + (CBS+TVM) on Chr1 of NA12878 (35×) is compared with three popular DoC-based methods, *viz*., ReadDepth (RD) [13], Control-FREEC (CF) [7] and CNVnator [18]. The window size is fixed at 300 bp for all the tools (including *i*CopyDAV) and mappability threshold is set at 0.8 in RD, CF and *i*CopyDAV (there is no provision to filter reads based on mappability in CNVnator) and the results are summarized in Table 5.

**Table 5:** Comparison of CNVs detected in *i*CopyDAV (combined approach, median + (CBS +TVM)) with ReadDepth, Control-FREEC and CNVnator (Mth = 0.8, window size 300 bp).

	*i*CopyDAV	ReadDepth	Control-FREEC	CNVnator
Total (Size in Mb)	120 (0.6)	177 (6.4)	64 (25.2)	266 (27.6)
Total	120 (0.6)	177 (6.4)	63* (5.2)	265* (7.6)
Gain	70 (0.4)	108 (6.0)	51* (3.3)	76 (3.2)
Loss	50 (0.2)	69 (0.4)	12 (1.9)	189* (4.4)

*Numbers after removing large CNV spanning centromere region in the chromosome.

In Table 5, a large variation is observed in the number (63–265), size (0.6–7.6Mb) and type of CNVs predicted by the four DoC-based approaches. Additionally, CF and CNVnator reported a large CNV event of 18 Mb at the locus chr1:123Mb-141Mb that overlaps with the centromere region, CF reported it as a ‘gain’ while CNVnator reported it as a ‘loss’. In ReadDepth and *i*CopyDAV this region is filtered out in the pre-treatment step as ‘unmappable’ region, and we do not consider this 18 Mb region for the comparative analysis. We also observe that large number (108) and size (6 Mb) of copy gain events are detected by ReadDepth, while CNVnator results in a large number of copy loss events (189) with a total span of 4.4 Mb. The number of CNVs detected by CF is low (especially copy loss, ~ 12) compared to the other DoC approaches, though the span is much larger (1.9 Mb) compared to that in RD and *i*CopyDAV. The large number of CNVs observed in CNVnator is due to no option of filtering low mappable regions (~ 204 of the predicted CNVs have an average mappability value < 0.8). On the other hand, the low number of CNVs predicted using CF is likely to be due to narrow range of GC-rich regions considered (> 0.35 and < 0.55). It is observed that ~ 18–23 CNVs predicted by other DoC-based methods fall in regions with GC content < 0.35 and > 0.55.

**Fig 10:**
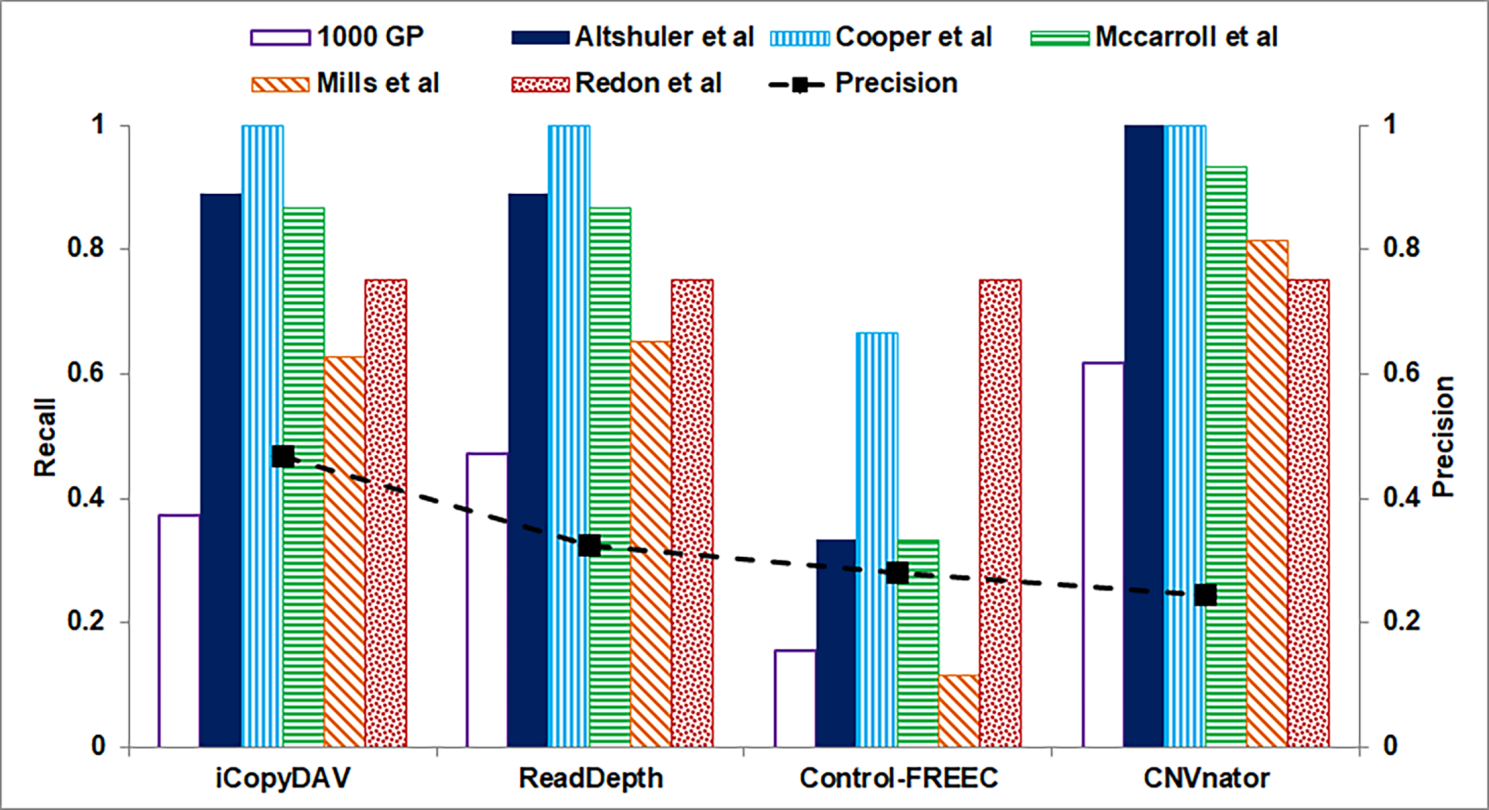
Performance of *i*CopyDAV with three DoC-based methods, ReadDepth, Control-FREEC, and CNVnator (using default parameters) is shown for high sequence coverage data (35×) of Chr 1 of NA12878 sample.

In Fig 10 is depicted the performance of four DoC based approaches with the six studies reported in DGV. We observe that CNVnator exhibits high recall values for all the six studies, however the precision is the least in this case (~ 0.25), indicating that majority of its predictions are false positives. It depicts high recall with 1000 GP compared to the other methods, possibly because CNVnator was one of the methods considered for annotation in this study. The high precision value of *i*CopyDAV and recall values comparable with ReadDepth (and only slightly lower compared to CNVnator), clearly indicates the reliability of CNV predictions by *i*CopyDAV. Since the six studies reported in DGV are not truly gold standard, it is possible that the CNVs predicted by *i*CopyDAV but not reported in any of the six studies may be true novel CNVs. An analysis revealed 28 CNVs detected by *i*CopyDAV that are not reported in the six DGV studies. When compared with the other three DoC methods, all of them were detected by at least one of the other three DoC methods and four CNVs (copy gain) are detected by all the four methods (S5 Table). A preliminary analysis of these four CNVs reveals that two of these CNVs at locus Chr1:14323800–143258700 and one CNV at Chr1:146173200–146197800 span the NBPF genes cluster, which is known to have evolutionary significance (discussed below in functional annotation analysis), but is not reported in DGV for NA12878 sample. The fourth CNV at Chr1:85752900–85778400 span the gene DDAH1, a key regulator in the breakdown of asymmetric dimethylarginine (ADMA). It has been shown that raised levels of ADMA are strongly correlated with increased risk of cardiovascular diseases [81].

**Computation time:** The *i*CopyDAV platform is computationally very efficient and can even be run on a desktop. The entire pipeline (data pre-treatment, variant calling and annotation steps) on Chromosome 1 of high coverage data (35×) took ~ 14 minutes CPU time on a 32 AMD Opteron with 2.4 GHz machine. Data pre-treatment step (computing read depth, filtering low mappable regions and correcting for GC bias) is computationally the most demanding step, requiring about ~ 10 minutes, while segmentation and variant calling steps took about ~ 4 minutes and the other steps just a few seconds. On the same machine the processing time required by ReadDepth and CNVnator is ~ 110 minutes and 130 minutes, respectively (~ 8–9×), while the time taken by Control-FREEC is comparable, ~ 15 minutes. We recommend a minimum requirement of 4 CPU machine running at 1.6 GHz for efficient performance of *i*CopyDAV.

**Fig 11:**
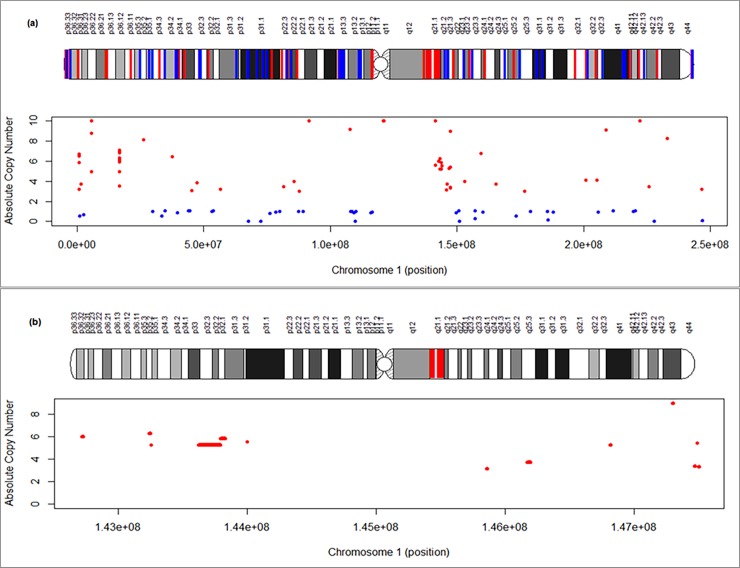
The location of CNVs, gain in ‘red’ and loss in ‘blue’ is shown (a) along Chr 1 of NA12878 sample, and (b) at the locus 1q21.1 spanning NBPF gene-family, which is rich in segmental duplications.

**Visualization and functional interpretation of predicted CNVs:** The distribution of CNVs along the chromosome can give insights about CNV-enriched regions, and association with other functional elements such as protein-coding genes, etc. which are likely to have phenotypic characteristics. In *i*CopyDAV, using the function ***plot***, the distribution of predicted CNVs can be visualized along the length of the chromosome or any region of interest (user-defined coordinates). The CNVs obtained using (TVM+CBS) approach for chromosome 1 of NA12878 sample on Median GC bias corrected data and mappability threshold, Mth = 0.8 are shown in Fig 11. A high resolution graph displaying copy gain events in ‘red’ and copy loss events in ‘blue’ on an ideogram (upper) and a 2d-plot (lower) is generated as shown in Fig 11(A). In Fig 11(B) the distribution of the CNVs at the locus 1q21.1 (Chr1:142,400,001–148,000,000) clearly indicates a cluster of CNVs encompassing NBPF-gene family, enriched with segmental duplications.

To enable the users to analyze the biological significance of predicted CNVs, functional and structural annotations are provided by the function ***annotate*** in *i*CopyDAV. Four categories of annotations are reported, *viz*., functional elements (genes, exons, promoters, lncRNAs, miRNAs, etc.), clinical relevance, structural features (segmental duplications, tandem repeats, etc.) and mapping to known CNVs reported in Database of Genetic Variants (DGV). Below we briefly discuss the functional analysis of CNVs obtained by Median+ (TVM+CBS) approach (Mth = 0.8) on using this module. The complete list of CNVs detected on Chr 1 is given in S6 Table along with annotations.

**a. Functional elements annotation:** Using the function ***annotate***, we identified 46 protein-coding genes (26 gain and 20 loss) mapping to 54 CNVs (35 copy gain and 19 copy loss events). To understand the role of these CNV-genes and identify any genotypic-phenotypic characteristics associated with Caucasian population, we carried out a systematic analysis of these genes. Copy loss of the Late Cornified Envelop-gene cluster (LCE1D, LCE1E, LCE3B, LCE3C and LCE3D) at locus 1q21.3 that is known to be part of epidermal differentiation complex is observed. These are involved in keratinization which in turn effects the melanosome distribution. In a study by de Cid et al [82], copy deletion of LCE3B and LCE3C is shown to be a risk factor for psoriasis in Western European patients. Copy loss of gene BMP8A at 1p34.2 is associated with reduced risk of Ankylosing Spondilytis in Korean populations [83]. Other functional elements encompassing the CNVs include eight long non-coding RNA (lncRNAs), TCONS_l2_00001057, TCONS_l2_00001060, TCONS_l2_00001061, TCONS_00001651, TCONS_00001652, TCONS_00002569, TCONS_00001785 and TCONS_00000355, spanning 5 CNVs (all gain) at loci 1p36.13 and 1q32.1. The functional significance of these lncRNAs is unknown, however, lncRNAs and their interaction with miRNA obtained from lncBase [84] reveal that 3 lncRNAs, TCONS_l2_00001060, TCONS_l2_00001061 and TCONS_00002569 are associated with risk of Breast neoplasm. One enhancer (element_809) spanning copy loss at 1p22.3 is observed in the vicinity of CNV-gene LMO4, which acts as a cell cycle regulator. Copy number variation of miRNA is known to affect the binding and regulation of miRNA target genes involved in various biological processes and may contribute to lethal diseases including cancer. We identified one miRNA-gene, MIR4654, spanning copy loss at 1q23.3 and three miRNA target sites (mir-368, mir-23 and mir-1/206) spanning 3′ UTR of CNV-gene SEC22B at 1q21.1 (gain). Gene SEC22B, regulated by mir-206, has been identified to play a role in the progression and development of Alzheimer’s disease [85]. Thus, we see that a systematic analysis of the functional regions overlapping with CNV predictions can help in identifying predisposition to diseases and in carrying out genotype-phenotype association studies.

**b. Clinical relevance:** For identifying the clinical relevance of CNV-genes, information from four resources, *viz*., ClinVar, OMIM, ExAC and DECIPHER, is collated and provided in *i*CopyDAV. The annotation results from these four resources for Chr 1 of NA12878 sample is summarized in the S6 Table. ClinVar reported three CNVs at loci 1q21.1 (2 copy gain events) and one at locus 1q41 (copy loss) to be ‘Pathogenic’. From literature review it is found that autism spectrum disorders are implicated with gain at 1q21.1 (5100 bp), and Loeys-Dietz syndrome associated with copy loss at 1q41 (600 bp). We also observe 26 CNVs identified at locus 1p36 characterized as ‘uncertain significance’ and association to Ductal Breast Carcinoma (DBC) phenotype from ClinVar. These CNVs (copy gain) span six genes CDK11A and CDK11B, NBPF1, MST1P2, CROCCP2 and ESPNP genes, of which the last three are pseudogenes. Two CDK family genes, CDK11A and CDK11B, are known to play multiple roles in cell cycle progression, cytokinesis and apoptosis, while the gene NBPF1 acts as a tumor suppressor by arresting cell cycle at G1 stage in Neuroblastoma [86]. Six copy loss genes (ST3GAL3, VANG, SLC1A7, LMO4, ERI3, XPR1) exhibit genic intolerance score > 0 in ExAC database, of which three genes (LMO4, ERI3 and XPR1) are also characterized as intolerant with haploinsufficient score ranging from 0–25% in DECIPHER. This implicates possible deleterious effect of copy variation of these genes. For instance, gene LMO4 (1p22.3) is a cell cycle transcriptor regulator and plays an important role in controlling cell cycle and cell proliferation [87]. The under-expression of this gene is linked with reduced breast cancer cell proliferation and cell cycle arrest at G2/M stage [87]. A CNV is found to be spanning intron-2 of the gene XPR1 (1q25.3), which is known to play a role in phosphate homeostasis, mediating phosphate export from the cell. Another four copy loss genes (VAV3, WDR47, NOS1AP and MSH4) are reported to have a haploinsufficient score < 25%, indicating these copy deletions may be pathogenic. Copy deletions are observed in the intronic regions for 9 of these haploinsufficient genes (except LMO4), however, their effect on the phenotype is not yet known. In recent studies, it has been shown that CNVs in introns regulate the expression of the genes directly through change in the intronic length, or variations in the regulatory regions (enchancers or CTCF binding sites) and non-coding functional RNA genes spanning them.

**c. Structural annotation:** About ~ 36% of predicted CNVs (42 copy gain and 2 copy loss) are observed to be overlapping with segmental duplications (SD) on Chr 1 of NA12878 Caucasian sample. The CNVs in the vicinity of SDs indicate their mechanism of formation via Non-allelic Homologous Recombination (NAHR). NAHR occurs by alignment followed by crossing over between two non-allelic, highly similar DNA sequences (repeats). Repeats on the same chromosome and in the same orientation mediate a duplication and/or deletion. In accordance with previous studies [88,89], NBPF gene-cluster spanning SD segments at 1q21.1 locus is identified: NBPF8 (gain: 3.7), NBPF10 (gain: 3.1), NBPF12 (gain: 5.2), NBPF20 (gain: 5.8) and NBPF25P (gain: 5.3), shown in Fig 11(B). These CNV-genes are reported to be formed as a consequence of human-lineage specific copy amplification which occurred about ~100 million years ago. The CNV-gene, NBPF1 (gain: 5.9) spanning 1p36.2 is a known primate-specific amplification, a common copy gain event observed in human and apes. These recently evolved NBPF gene clusters are characterized by highly conserved domains of DUF1220. O’Blenesis et al [89] showed a high copy number of DUF1220 protein domain in humans (272 copies) compared to chimpanzee (90–125 copies) and similar numbers are observed in our analysis. These amplifications are fixed in the population as a result of positive selection and have been identified to contribute in neuron number, brain size and cognitive ability that occurred specifically in humans (and other primates) [90]. Low copy number of DUF1220 is shown to result in brain growth abnormalities, leading to microencephaly, while high copy numbers of the domains are associated with macroencephaly [91]. A number of CNVs are also observed to be overlapping with tandem and interspersed repeats: 9 copy gain and 5 copy loss events spanning tandem repeats, with an average of 4 tandem repeats per CNV (range: 1–32), and 5 copy gain and 4 copy loss events have their breakpoints overlapping the interspersed repeats, with 1 CNV overlapping Long terminal repeat retrotransposons (LTR) and the rest with LINEs (listed in S6 Table). Six out of nine copy gain events at Chr1:121053000–121055700, Chr1:121056900–121057500, Chr1:121185900–121186800, Chr1:147299700–147302400, Chr1:152953200–152953800 and Chr1:222266700–222268800 have a > 90% overlap with tandem repeats and two copy gain events at Chr1:5657700–5658600 and Chr1:146816400–146819400 have an overlap > 90% with LINES. Thus, the extent of overlap with other known structural variants can help in understanding the mechanism of the predicted CNV (partial overlap at breakpoints), or a common CNV fixed in population (> 90% overlap).

**d. Known CNVs:** After discarding CNVs having > 90% overlap with other structural variants based on our structural annotation analysis, we have a total of 112 CNVs predicted on Chr 1 of NA12878 Caucasian sample. Of these, 106 (~ 95%) are reported in Database of Genomic Variants (DGV) on considering ≥ 50% overlap criteria. The six CNVs which are not reported in DGV include two copy gain (Chr1:45248700–45249300 and Chr1:81627600–81630300) and four copy loss (Chr1:44519700–44520300, Chr1:87567000–87567900 (spanning LMO4 gene), Chr1:211583100–211583700 and Chr1:219713700–219714300), spanning a total of 6600 bp. Since the majority of CNVs reported in DGV are obtained from array-based techniques, these smaller CNVs, ranging from 600 bp to 2700 bp are likely to be missed by these approaches. Among these six CNVs, copy gain at Chr1:81627600–81630300 (span intron-1 of gene LPHN2) and copy loss at Chr1:211583100–211583700 are also identified as copy variant regions by ReadDepth and CNVnator, respectively.

Thus, using the annotation module in *i*CopyDAV, we identified 43 CNVs that may be of clinical importance (high priority) and 36 CNVs that may have certain effect on gene expression (medium priority) (see S6 Table). It may be noted that ~ 70% of these CNVs are also predicted by at least one of the three other DoC-based methods (ReadDepth, CF and CNVnator), and ~ 94% also reported in DGV, indicating the reliability of *i*CopyDAV in identifying and categorizing the variants.

## Discussion and conclusion

Copy number variation is implicated in numerous complex diseases. A large number of methods have been proposed for CNV detection using NGS data to carry out genome-wide association studies. However, it is not being used to its full potential due to number of issues associated with NGS based CNV detection. Comparative analysis of various CNV detection tools have shown major differences in the type, size and copy number of the CNVs predicted raising concerns about the validity of the results. To understand the discrepancy in the results across various tools, in this study we analyze the impact of different data pre-treatment options, bin size and sequencing depth on the segmentation approaches and breakpoint accuracy. The analysis is carried out using *i*CopyDAV, an integrated platform developed by us for CNV detection in whole genome NGS data using DoC-based approaches.

In the DoC-based approaches for CNV detection, to handle non-uniform distribution of reads along the genome, data pre-treatment step is very crucial in accurate detection of absolute copy number of the variant regions, and in reducing false positives and false negatives. Two major steps in data pre-treatment involve correcting for mappability bias and GC bias. Here we show that methods for the correction of these two biases greatly affect the number and type of copy variants detected and these should be judiciously chosen depending on the sequencing depth and the segmentation method used. We present our results for two threshold values for filtering of low mappability reads (Mth = 0.5 and 0.8) and two methods for removing GC bias based on normalization (Median) and regression (Loess) approach, respectively on two segmentation methods, one divisive (CBS) and the other agglomerative (TVM) approach. The major results of our analysis are summarized in Table 6.

**Table 6:** Comparative analysis of various parameters affecting variant calling in TVM and CBS segmentation approaches.

**Segmentation Method: TVM**	
Bin size	Small, no dependence on sequencing depth
Breakpoint error	Small
Sequencing depth (6×→ 35×)	Reduction in CNVs predicted on increasing sequencing depth (both gain and loss), No significant difference in gain events for any data pre-treatments
Mappability cutoff (0.5→0.8)	Loss (reduction), Gain (increase) in Low coverage, no significant difference in High coverage
Loess *vs* Median	Reduction in no. of CNVs predicted with Median, No Loss events (low coverage)
No GC *vs* GC bias corrected data	No difference in case of High coverage (Mth = 0.8), indicating no need for GC bias correction in this case
**Segmentation Method: CBS**	
Bin size	Dependent on sequencing depth, requires larger bin sizes at low coverage
Breakpoint error	Large, dependent on sequencing depth
Sequencing depth (6×→ 35×)	Large reduction in Gain events (from low to high coverage)
Mappability cutoff (0.5→0.8)	Large reduction in Loss events (for No GC, Loess), No difference (Median)
Loess *vs* Median	Large reduction in number of CNVs predicted (Loess), No difference (Median)
No GC *vs* GC bias corrected data	Large difference observed, clearly indicating the need for GC bias correction

A marked difference in CNV predictions by the two segmentation approaches is observed, especially in low coverage data. We note that mappability threshold value is dependent on the sequencing depth, and lower cutoff (Mth = 0.5) results in optimal performance in low coverage data (6×), while high coverage data (35×) requires a larger cutoff value of Mth = 0.8 for accurate detection of CNVs. Also, in general, lower numbers of CNV predictions are obtained on using TVM segmentation approach compared to CBS approach for a given value of Mth and sequencing depth. The two methods for GC bias correction also show a clear dependence on the sequencing depth and segmentation approach used. For instance, in low sequence coverage data, Median GC corrected data resulted in negligibly small number of copy loss events (with both the segmentation approaches), irrespective of the mappability threshold value. This indicates that Median approach is not suitable for detection of copy loss events in low coverage data. Also, in high coverage data, reduction in the copy gain events is observed with both the GC bias correction methods (higher in Loess corrected data), compared to in low coverage data. We observe that different combinations of data pre-treatment and segmentation methods show varied performance in the detection of different type and size of CNVs. For example, Loess+TVM gives the most reliable results in low coverage data (with Mth = 0.5 or 0.8), while Median+TVM performs better in high coverage data when Mth = 0.5. When Mth = 0.8, both Median+CBS and Loess+TVM result in reliable predictions. These results clearly indicate the inter-dependence of data pre-treatment and segmentation methods.

Apart from data pre-treatment, we show that bin size and sequencing depth are two other very crucial parameters that affect the CNVs detected in a given segmentation method. For optimal performance, bin size needs to be appropriately chosen according to the coverage of the data and the segmentation method. It is noted that very large bin sizes are required for low coverage data with CBS segmentation approach (also ReadDepth and Control-FREEC). No such dependency is observed with TVM approach, and a small bin size (~ 100 bases) can result in accurate detection even in low sequence coverage data in this case. Since smaller bin sizes result in smaller breakpoint error, this makes TVM the method of choice in the case of low coverage data. From our simulated experiment, we observe that for the detection of CNVs ≥ 500 bases, the performance of *i*CopyDAV (TVM) is reliable, with an F-score of ~ 0.9 or higher at 30× sequencing coverage. Most CNV detection approaches have shown similar performance but in the detection of very large CNVs (> 10Kb). This makes *i*CopyDAV an attractive CNV tool for the detection of small CNVs, especially with customizable data pre-treatment options. We also observe that larger CNVs (> 5Kb) are easily detected compared to smaller CNVs in low sequence coverage data, but no such dependence is observed in high sequence coverage data. Improved performance is also observed for the detection of both small and large copy gain and copy loss events with increase in sequencing depth. *i*CopyDAV is under active development and we anticipate incorporating additional features to extract paired-end information (and implement split-read based approaches). This would help in refining the breakpoint accuracy to 1bp and also enable detection of very small CNVs (< 500 bases) accurately. We have shown our results on both simulated and real data and observe consistency in the predictions for various combinations studied. We hope that the suggestions of this study would aid the researchers in making appropriate choices in the data pre-treatment step in using DoC-based approaches for CNV detection.

The advantage of an integrated platform such as *i*CopyDAV allows the user to customize the workflow for CNV detection depending on the type and size of CNVs to be detected and the sequencing depth. It also allows easy integration of results from different workflows within the same platform to detect a wider spectrum of CNVs. We observe that sensitivity (recall) in identifying true CNVs is higher on using CBS approach, while precision is higher with TVM suggesting that the overall performance can be improved by combining the results of the two approaches. Also, detection of duplications using TVM approach shows higher precision compared to deletions, as observed in simulated experiment. Thus, combining predictions from the two approaches lead to an improved performance compared to the individual methods. Additionally, the advantage of Depth-of-coverage (DoC) based approaches implemented in *i*CopyDAV is that absolute copy number of the variant regions can be obtained.

*i*CopyDAV provides more functionalities than other tools. It can handle CNV detection in both single as well as paired-end whole genome NGS data (though currently it does not use the paired-end information) and also when no control sample is available. An important feature of *i*CopyDAV is the visualization module that helps the user to generate high quality CNV distribution plots along the chromosome or user defined coordinates, and on the ideogram. The annotation module in *i*CopyDAV aids in functional, structural and evolutionary analysis of predicted CNVs and prioritize the CNVs based on their functional and clinical relevance. It is accurate (> 95% agreement with DGV annotations) and is computationally very efficient and takes only a couple of minutes to scan a complete chromosome due to the parallel implementation of the segmentation approaches. It is freely available and can be easily downloaded as Docker’s image and is currently compatible with two widely used human reference genome assemblies, hg18 and hg19. *i*CopyDAV is a unique pipeline providing a complete end-to-end solution from data pre-treatment to annotation and visualization of CNVs. The modular framework of *i*CopyDAV has been designed with the anticipation to extend *i*CopyDAV to detection of other DNA variants, namely, single nucleotide variations (SNVs), insertions and deletions (indels), etc. and also incorporate paired-end and split-read based strategies to further improve the resolution of CNV boundaries.

## Supporting information

S1 Fig GC profile of reads before (red) and after (blue) GC correction using Loess regression implemented in *i*CopyDAV. (TIF)

S2 Fig Performance of TVM (depicted by ‘circle’) and CBS (depicted by ‘triangle’) on using window/bin size as 50 bp (‘open’ symbol) and using optimal bin size (‘closed’ symbol) in simulated data. (TIF)

S3 Fig (a) Recall and (b) Precision of DoC-based tools to identify small (≤ 1 Kb) and large CNV (> 1 Kb) indicated in ‘solid’ and ‘dashed’ lines respectively, in simulated data.
(TIF)

S4 Fig (a) Recall and (b) Precision of DoC-based tools to identify all sizes of CNVs in simulated data.
(TIF)

S5 Fig Performance of *i*CopyDAV (TVM, CBS and their combination) and comparison with other DoC based tools to identify small (≤ 1 Kb) and large CNV (> 1 Kb) indicated in ‘solid’ and ‘dashed’ lines respectively, in simulated data. (TIF)

S1 Table Loci of various CNVs inserted in position Chr4:75,671,302–115,671,302 (40 MB) of hg18 reference assembly for generating simulated data (coordinates with respect to ‘reference’ sequence not reference genome assembly). (TIF)

S2 Table List of studies considered for benchmark for CNVs predicted in Chr1 of NA12878 samples. All CNVs listed in these six studies are extracted for Chromosome 1 of NA12878 sample that are of size ≥ 600 bp (2 bin size), mappability ≥ 0.5 and are mapped to hg18 human reference genome.
(TIF)

S3 Table Summary of CNVs predicted in Chr1 of NA12878 for low sequence coverage data (6×) using various combinations of GC correction and mappability thresholds. (TIF)

S4 Table Summary of CNVs predicted in Chr1 of NA12878 for high sequence coverage data (35×) using various combinations of GC correction and mappability thresholds. (TIF)

S5 Table CNVs predicted by *i*CopyDAV (Combined set of Median+(TVM+CBS)) that overlap with CNVs predicted by other DoC-based methods but do not overlap with any annotations for Chr1 of NA12878 sample considered for benchmark. (TIF)

S6 Table List of CNVs identified in Chr 1 of NA12878 using *i*CopyDAV and their annotations (SD- segmental duplications, LINE- Long Interspersed Nuclear Elements, LTR- Long Terminal Repeat Retrotransposons, ‘*’—ClinVar, ‘†’–DECIPHER Haploinsufficiency score (0–25% HI score, reported for copy loss), ‘Ψ’–ExAC genic intolerance (score > 0, reported for copy loss), DBC- Ductal Breast Carcinoma). (XLSX)
