# Supplementary figures and images for: iCopyDAV: Integrated platform for copy number variations—Detection, annotation and visualization
Source: PLoS One. 2018 Apr 5;13(4):e0195334. doi: 10.1371/journal.pone.0195334 (PMC5886540; doi:10.1371/journal.pone.0195334)

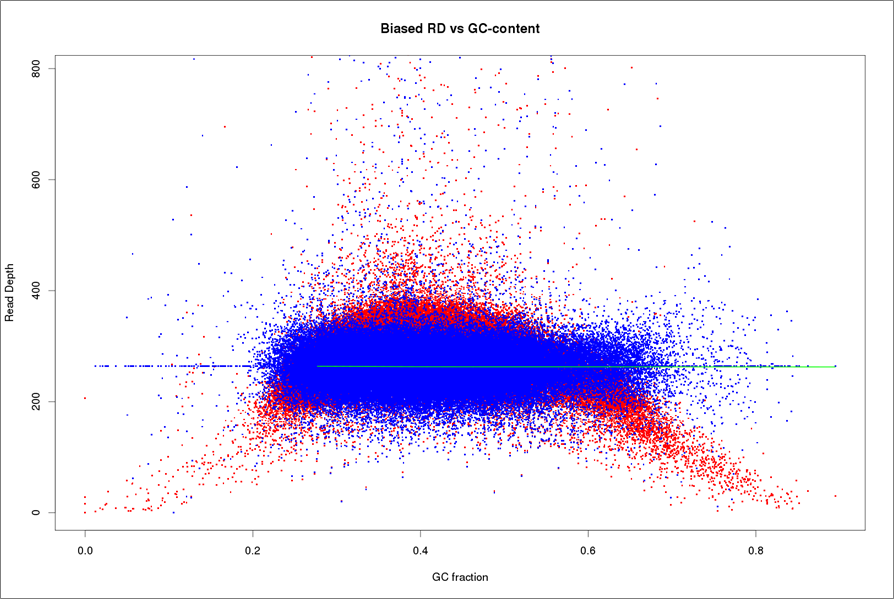

Supplement: S1 Fig — (TIF) [file pone.0195334.s001.tif]

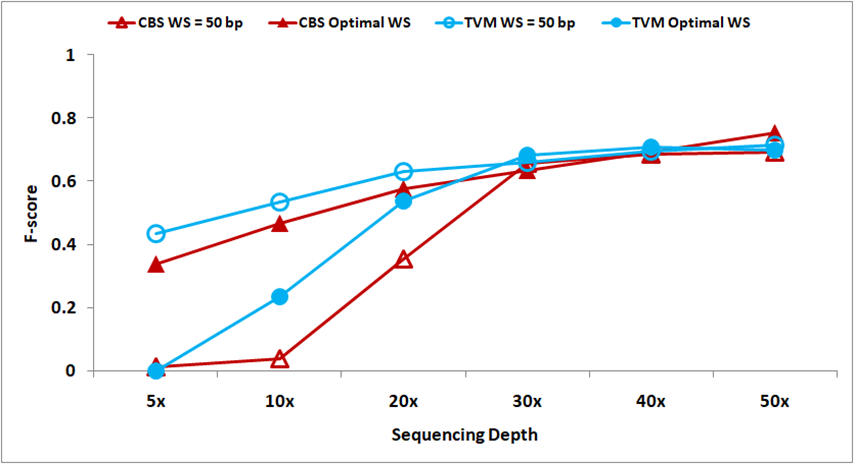

Supplement: S2 Fig — (TIF) [file pone.0195334.s002.tif]

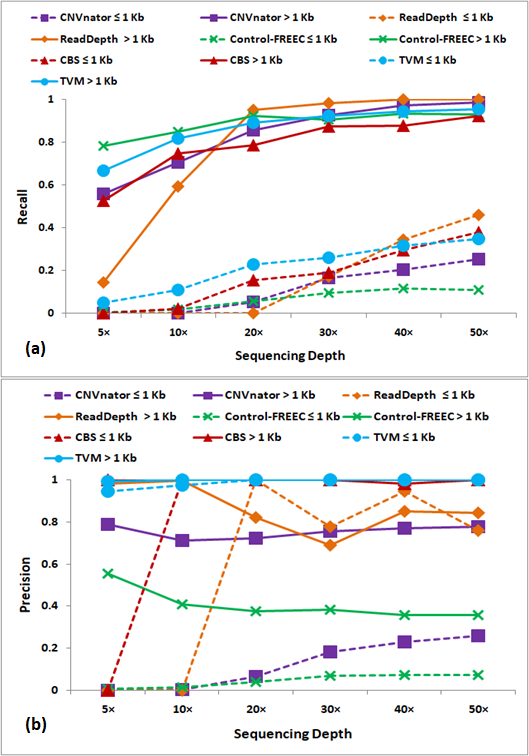

Supplement: S3 Fig — (a) Recall and (b) Precision of DoC-based tools to identify small (≤ 1 Kb) and large CNV (> 1 Kb) indicated in ‘solid’ and ‘dashed’ lines respectively, in simulated data. (TIF) [file pone.0195334.s003.tif]

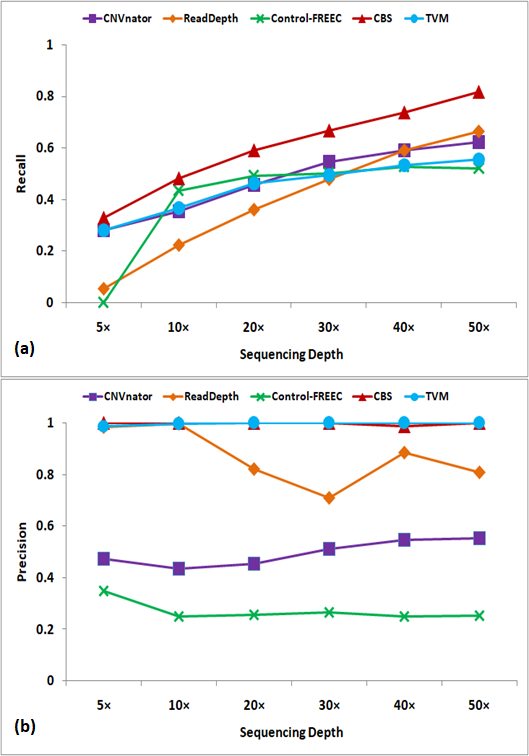

Supplement: S4 Fig — (a) Recall and (b) Precision of DoC-based tools to identify all sizes of CNVs in simulated data. (TIF) [file pone.0195334.s004.tif]

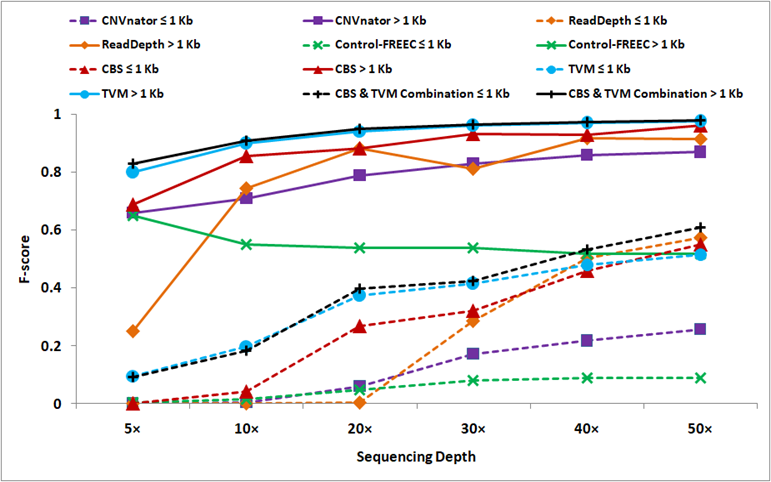

Supplement: S5 Fig — (TIF) [file pone.0195334.s005.tif]

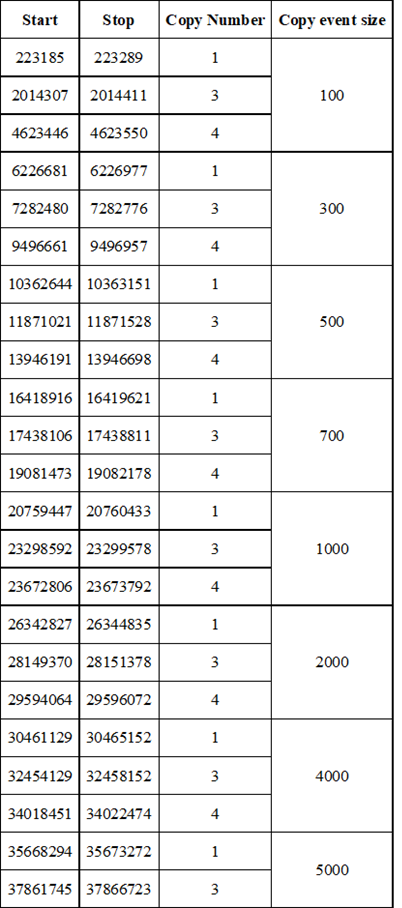

Supplement: S1 Table — (TIF) [file pone.0195334.s006.tif]

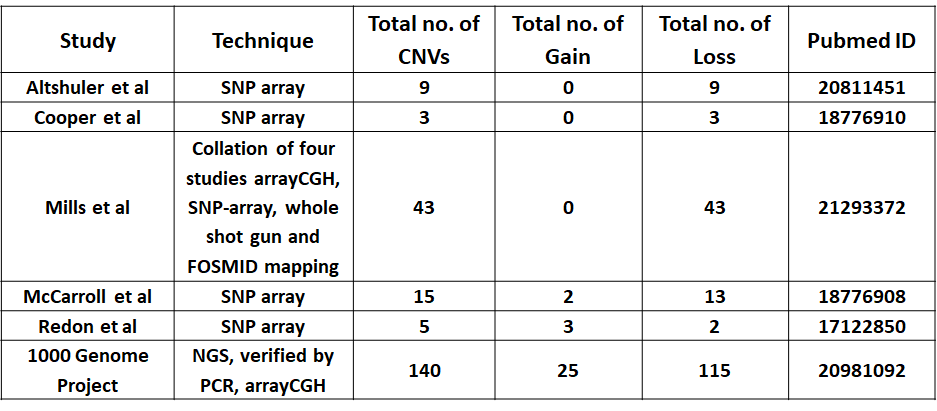

Supplement: S2 Table — All CNVs listed in these six studies are extracted for Chromosome 1 of NA12878 sample that are of size ≥ 600 bp (2 bin size), mappability ≥ 0.5 and are mapped to hg18 human reference genome. (TIF) [file pone.0195334.s007.tif]

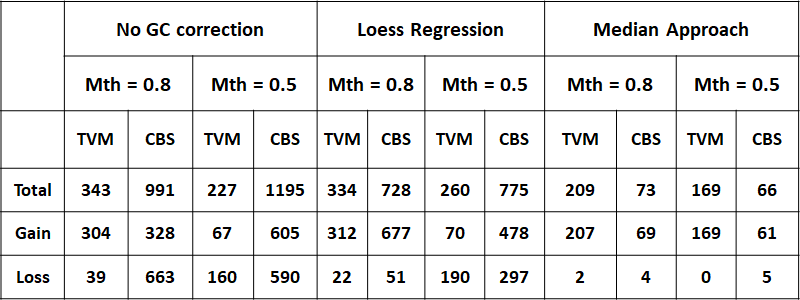

Supplement: S3 Table — (TIF) [file pone.0195334.s008.tif]

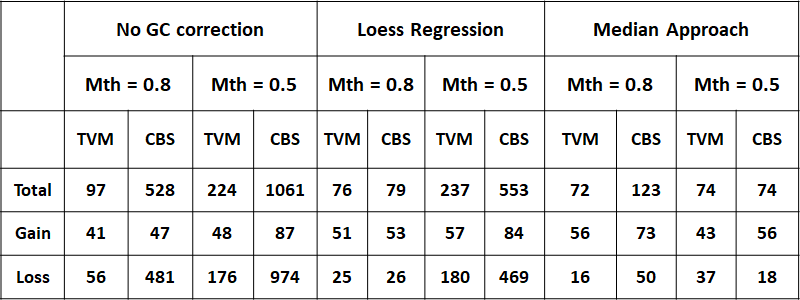

Supplement: S4 Table — (TIF) [file pone.0195334.s009.tif]

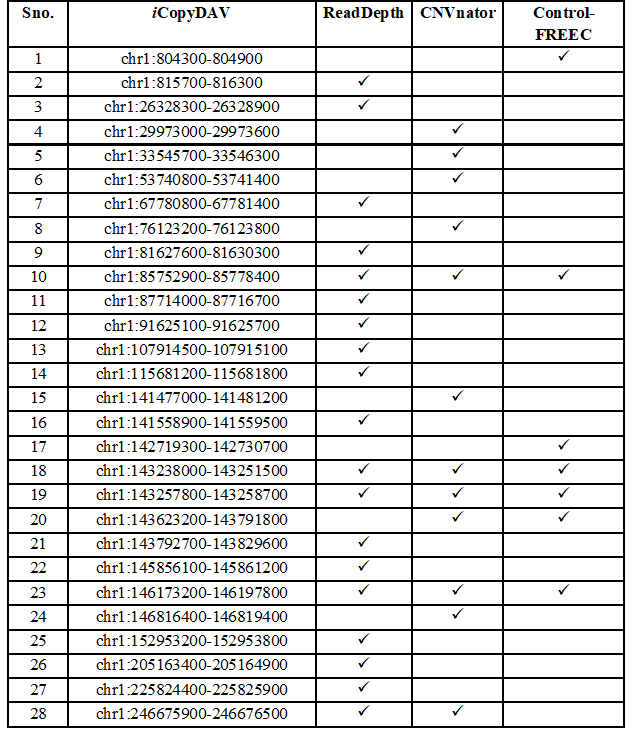

Supplement: S5 Table — (TIF) [file pone.0195334.s010.tif]
